# Exosomal HSPB1, interacting with FUS protein, suppresses hypoxia‐induced ferroptosis in pancreatic cancer by stabilizing Nrf2 mRNA and repressing P450

**DOI:** 10.1111/jcmm.18209

**Published:** 2024-04-29

**Authors:** Lun Zhang, Liuxu Yang, Keyuan Du

**Affiliations:** ^1^ Department of Hepatobiliary Surgery The First Affiliated Hospital of Xi'an Jiaotong University Xi'an Shaanxi P.R. China; ^2^ Health Science Center Xi'an Jiaotong University Xi'an Shaanxi P.R. China

**Keywords:** exosomal HSPB1, ferroptosis, FUS, Nrf2/HO‐1/P450, pancreatic cancer

## Abstract

Ferroptosis is a new type of programmed cell death, which has been involved in the progression of tumours. However, the regulatory network of ferroptosis in pancreatic cancer is still largely unknown. Here, using datasets from GEO and TCGA, we screened HSPB1, related to the P450 monooxygenase signalling, a fuel of ferroptosis, to be a candidate gene for regulating pancreatic cancer cell ferroptosis. We found that HSPB1 was enriched in the exosomes derived from human pancreatic cancer cell lines SW1990 and Panc‐1. Then, hypoxic SW1990 cells were incubated with exosomes alone or together with HSPB1 siRNA (si‐HSPB1), and we observed that exosomes promoted cell proliferation and invasion and suppressed ferroptosis, which was reversed by si‐HSPB1. Moreover, we found a potential binding affinity between HSPB1 and FUS, verified their protein interaction by using dual‐colour fluorescence colocalization and co‐IP assays, and demonstrated the promoting effect of FUS on oxidative stress and ferroptosis in hypoxic SW1990 cells. Subsequently, FUS was demonstrated to bind with and stabilize the mRNA of Nrf2, a famous anti‐ferroptosis gene that negatively regulates the level of P450. Furthermore, overexpressing FUS and activating the Nrf2/HO‐1 pathway (using NK‐252) both reversed the inhibitory effect of si‐HSPB1 on exosome functions. Finally, our in vivo studies showed that exosome administration promote tumour growth in nude mice of xenotransplantation, which was able to be eliminated by knockdown of HSPB1. In conclusion, exosomal HSPB1 interacts with the RNA binding protein FUS and decreases FUS‐mediated stability of Nrf2 mRNA, thus suppressing hypoxia‐induced ferroptosis in pancreatic cancer.

## 1 INTRODUCTION

Pancreatic cancer is currently one of the cancers with the highest mortality and lowest survival rate of all. With a small size, located in the retroperitoneum of the human upper abdomen, the pancreas is a kind of gland that has both endocrine and exocrine functions, and its pathological alterations and physiological effects have an important impact on the life health of people. It was reported that pancreatic cancer in the United States ranks 4th in cancer death and has a 5‐year survival rate of less than 5%.^1^ Data from the National Cancer Registry indicates that the number of cases and deaths from pancreatic cancer in China in 2014 was about 922,000 and 811,000, respectively, ranking at 10th and 6th in terms of cancer incidence and death.^2^

Iron ions are important catalysts of lipid peroxidation, and their uptake, release and storage in cells are important in cell metabolism and death. Inhibition of the cysteine desulfurase nitrogen fixation 1 homologue (NFS1), which provides sulphur from cysteine for the synthesis of iron‐sulphur clusters, activates an iron starvation response by simultaneously increasing TFRC (transferrin receptor protein 1) expression and degrading FTH1 (ferritin heavy chain 1), causing increased free iron, thereby sensitizing cells to iron‐depended death.^3^ Recent studies have shown that ferroptosis displayed great potential in the treatment of many solid tumours, including breast, kidney, lung, pancreas, liver, and head and neck cancers. Traditional cell death modalities include apoptosis, autophagic death, and necrosis, and have their specificity in the mechanism of occurrence, morphological features, and biological functions. Unlike traditional cell death modalities, ferroptosis is an iron‐ and lipid peroxidation‐dependent form of cell death, the main cytological changes of which are characterized by the loss or disappearance of mitochondrial cristae, rupture of the outer mitochondrial membrane and mitochondrial membrane shrinkage caused by the peroxidation of lipid components of the cell membrane and loss of selective permeability of the cell membranes due to oxidative stress.^4^, ^5^ Genes such as nuclear receptor coactivator (Nrf2) and p53 have been found to influence the sensitivity of cells to ferroptosis by regulating intracellular iron and lipid peroxide metabolism. In addition, glutathione peroxidase‐4 (GPX4), which can reduce the production of reactive oxygen species (ROS) and inhibit lipid peroxidation under the influence of the prosthetic group glutathione (GSH), plays a suppressive role in ferroptosis in tumour cells.^6^ However, the regulatory network of ferroptosis in pancreatic cancer is still largely unknown.

During the development and metastatic progression of the tumour, tumour cells and the surrounding microenvironment mutually influence each other, through directly interacting or mediated by secreting cytokines or extracellular vesicles. Exosomes are a form of extracellular vesicles, ranging in size from 40 to 100 nm in diameter and with a bilayer lipid structure, and are usually secreted and released by a variety of cell types in many body fluids such as blood, saliva, and urine.^7^ One of the main functions of exosomes is to transport nucleic acids and proteins into various recipient cells, with important implications for intercellular material transport and information communication. Exosomes contain a variety of proteins, including members of the heat shock protein family, Tetraspanins (CD9, CD81, CD69), ESCRT‐related proteins (TSG101), cytoskeletal proteins (actin and tubulin) and GTPases, which are involved in the production and secretion of exosomes and also in physiological and biochemical processes such as antigen presentation, membrane microarchitecture, cytoskeleton.^8^ Studies have shown that exosomes play an important role in a variety of cancers including pancreatic cancer and may serve as stimulators of pancreatic cancer initiation and progression.

In this study, we searched potential ferroptosis regulatory genes by analysing datasets from the public databases Gene Expression Omnibus (GEO) and the Cancer Genome Atlas (TCGA), and six differentially expressed genes (DEGs) were screened to be potentially involved in the regulation of pancreatic cancer cell ferroptosis, among which HSPB1 is an exosomal protein and relatively less characterized regulator in pancreatic cancer. Moreover, the role and mechanism of exosomal HSPB1 in regulating pancreatic cancer cell ferroptosis was investigated in‐depth.

## 2 MATERIALS AND METHODS

### 2.1 Bioinformatics analyses for datasets from GEO and TCGA

The GSE157830 dataset was downloaded from the GEO database. Bioinformatics data analysis was provided by NewCore BioTech (Shanghai, China). Briefly, fastp software (v0.20.0) was used to trim adaptor and remove low‐quality reads to get high‐quality clean reads. STAR software (v2.7.9a) was used to align the high‐quality clean reads to the human reference genome (hg38). The featureCounts software (v2.0) was used to get the raw gene level mRNA read counts as the mRNA expression profile. DESeq2 software (v1.30.1) was used to normalize and calculate the fold change and P‐value between two groups. Ensembl GTF gene annotation database (v104) was used to annotate the mRNA. The rMATS software (v4.1.1) was used to predict the alternative splicing events between two groups.

The gene expression matrix of the TCGA‐PAAD project was downloaded from GDC using R package TCGAbiolinks, and gene expression differential analysis was conducted using DESeq2, comparing TP (Primary Solid Tumour) with NT (Solid Tissue Normal). A volcanic map was drawn using the R package ggplot2. A heat map of differentially expressed genes was drawn using the R package pheatmap. Use the R package ClusterProfiler to perform GO and KEGG enrichment analysis on differentially expressed genes (DEGs).

The online bioinformatic tool Jvenn was used to intersect DEGs among the GSE157830 dataset and TCGA‐PAAD project. Gene Ontology and KEGG pathway enrichment analysis were performed with the cluster Profiler R package (v3.18.1) based on the DEGs.

### 2.2 Cell culture

The human pancreatic cancer cell lines AsPC1, SW1990, BxPC‐3 and Panc‐1, and the normal human pancreatic ductal epithelial cell line HPDE6c7 were purchased from the National Collection of Authenticated Cell Cultures (Shanghai, China). The cells were incubated in a medium containing 10% foetal bovine serum and 100 U/mL penicillin and 100 μg/mL streptomycin (Sigma, St. Louis, MO, USA) in RPMI 1640 medium (Gibco, Rockville, MD) at 5% CO_2_ at 37°C. For mild hypoxia treatment, cells were cultured under an atmosphere of c5% O_2_–5% CO_2_. The pcDNA‐HSPB1, pcDNA‐FUS and their negative control (Vector) were purchased from RiboBio Co., Ltd (Guangzhou, China) and transfected using RiboBio Transfection Kit (RiboBio Co., Ltd). Small interfering RNA against HSPB1 (si‐HSPB1), FUS (si‐FUS) and their negative control (scramble) were designed and synthesized by Invitrogen (Carlsbad, CA, USA). All transfections were performed by using Lipofectamine 3000 Transfection Reagent (Invitrogen) according to the manufacturer's instructions.

### 2.3 Xenotransplantation of human pancreatic cancer in nude mice

Adult nude mice (6–8 weeks, 20–22 g, Wuhan Experimental Animal Center, Wuhan City, China) were housed in a specific pathogen‐free environment under the condition of 12 h light/12 h dark cycle, free access to food and water, and acclimatized to their surroundings for 7 days. 1 × 10^7^ SW1990 cells were subcutaneously injected into the armpit of nude mice to establish a xenotransplantation model of human pancreatic cancer. Xenotransplantation nude mice were randomly divided into three groups (*n* = 8 per group) including Scramble group (locally injected with lentiviral interference vector inserted with scrambled shRNA, weekly, 200 μL, 10^13^ PFU/mL), EXO group (locally injected with scrambled shRNA plus exosomes, weekly, 200 μL), and EXO+sh‐HSPB1 group (locally injected with exosomes plus scrambled shRNA, weekly, 200 μL, respectively). The tumour volume was closely observed and measured on days 7, 14, 21 28 and 35 after injection. On day 35, nude mice were euthanized with 150 mg/Kg pentobarbital sodium, and tumour tissues were collected for subsequent studies. This study was approved by the Ethics Committee of Xi'an JiaoTong University (XJTULAC‐2022021J).

### 2.4 Exosome extraction and verification

The supernatant of cell culture medium was collected, and the cell components and dead cells were removed by low‐speed centrifugation (300 *g*, 10 min, 2000 *g*, 10 min) at 4°C. The supernatant containing exosomes was retained and the cell debris was removed by high‐speed centrifugation (10,000 *g*, 70 min). The supernatant containing extracellular vesicles was retained and the exosomes were precipitated by ultracentrifugation (100,000 *g*, 70 min). Appropriate amount of PBS was taken to resuspend the extracellular vesicle precipitation and then ultracentrifuged again (100,000 *g*, 70 min) to eliminate contaminated proteins. The precipitation was collected and divided, and stored at −80°C for future use.

### 2.5 Reverse transcription‐quantitative PCR (RT‐qPCR)

Total RNA was extracted from cells using TRIzol reagent (Invitrogen, Carlsbad, CA, USA) at the instruction of manufacturer. Reverse transcription was performed by using a PrimeScript RT reagent Kit (Takara Biotechnology, Dalian, China). Real‐time PCR analysis was conducted with the SYBR Premix Ex Taq II (Takara, Dalian, China) on an ABI 7500 Real‐Time PCR System (Applied Biosystems, Foster City, CA) under the following conditions: 95°C for 1 min, and then 35 cycles at 95°C for 20 s, 56°C for 10 s and 72°C for 15 s. The primers used in this study were synthesized from Sangon Biotech (Shanghai, China), the sequences of which were as follows: HSPB1: 5′‐GCA CGG CTA CAT TTC CCG TTG CTT CAC‐3′, 5′‐TTA CTT GTT TTC CGG CTG TTC GGA CTT CCC‐3′; NRF2: 5′‐GAG GAT GGG AAA CCT TAC TTT‐3′, 5′‐ATA TTT GCA GTT GAA GGC CTT‐3′; β‐tubulin: 5′‐CGG TAA CAA CTG GGC CAA GG‐3′, 5′‐CGG TAA CAA CTG GGC CAA GG‐3′. The relative expression levels were normalized by using the 2^−ΔΔCt^ method.

### 2.6 Western blotting

Total protein was extracted from cells and tissues by using RIPA lysis buffer (Beyotime, Shanghai, China). Total protein concentration was measured by the BCA Protein Assay Kit (Beyotime, Shanghai, China). Then proteins were separated on SDS‐PAGE under the following conditions: 70 V for 30 min, followed by 120 V for 90 min. And then the protein bands were transferred onto polyvinylidene fluoride (PVDF) membranes (Millipore, Bedford, MA, USA) at 300 mA for 2 h. After being blocked with 5% nonfat milk for 2 h at room temperature, the membranes were incubated overnight at 4°C with the following primary antibodies: HSPB1 antibody (1:500, Abcam, ab62339), CD63 antibody (1:400, Abcam, ab134045), CD81 antibody (1:400, Abcam, ab109201), TSG101 antibody (1:300, Abcam, ab30871), Alix antibody (1:300, Abcam, ab117600), GXP4 antibody (1:1000, Abcam, ab75801), FTH1 antibody (1:500, Abcam, ab75972), Nrf2 antibody (1:300, Abcam, ab137550), HO‐1 antibody (1:400, Abcam, ab68477), and POR antibody (1:500, Abcam, ab257595), Cyb5r1 antibody (1:300, Abcam, ab104217) and β‐tubulin antibody (1:800, Abcam, ab176560). Then, the membranes were incubated with horseradish peroxidase (HRP)‐conjugated goat anti‐rabbit IgG (1:2000, Abcam, ab6721) at 37°C for 1 h. The protein bands were visualized by using the Enhanced chemiluminescence reagents (Millipore, MA, USA) in a Gel Imaging System (Thermo Fisher Scientific). The expression of the relative protein was evaluated by the grey value ratio of the target protein to the internal reference GAPDH and analysed with ImageJ software (Thermo Fisher Scientific).

### 2.7 CCK‐8 assay

SW1990 cells grown in the logarithmic phase were digested with 0.25% trypsin, the supernatant was discarded, and then the cell density was adjusted to 3 × 10^4^ cells/mL by adding DMEM medium. The cell suspension was seeded in 96‐well plates, and 100 μL was added into each well, and placed in an incubator at 37°C and 5% CO_2_ for 4 h. The absorbance at 450 nm was detected by microplate reader.

### 2.8 Transwell cell invasion assay

Cells were seeded into the upper chamber of Transwell chambers (8.0 μm pore size; Millipore Corporation, USA) pre‐coated with Matrigel (BD Bioscience, USA). The complete medium was added to the lower chamber. After incubation at 37°C for 48 h, cells in the upper chamber were removed with cotton swabs, and cells in the lower chamber were fixed with 70% ethanol and stained with 0.1% crystal violet. The invasive cells were counted under a light microscope (Olympus, Tokyo, Japan).

### 2.9 Co‐IP assay

Forty‐eight hours after transfection, the cells were washed twice with ice‐cold PBS and lysed with RIPA lysis buffer. The supernatant collected by centrifugation was incubated with corresponding antibodies at 4°C overnight. Then 100 μL of Protein A agarose beads were added to capture the antigen–antibody complex, and slowly shook the mixture at 4°C overnight. The agarose beads‐antigen–antibody complex was collected by instantaneous centrifugation, and washed with ice‐cold PBS. Then the complex was boiled with protein loading buffer to free the antigen, antibody and beads. After centrifugation, the supernatant was taken for electrophoresis to detect the expression of the interaction protein.

### 2.10 RNA immunoprecipitation (RNA‐IP)

Magna RIP RNA‐Binding Protein Immunoprecipitation Kit (Millipore) was used to verify the binding of FUS and NRF2 mRNA. In brief, 10^7^ cells were washed in ice‐cold PBS, lysed in 500 μL of RIP buffer and incubated with 5 μg of primary FUS antibody at 4°C for 2 h. A total of 40 μL of 50% slurry of protein A‐Sepharose was added to each sample, and the mixtures were incubated at 4°C for 4 h. The pellets were washed with PBS and resuspended in 0.5 mL of Tri Reagent. The coprecipitated RNA eluted in an aqueous solution was analysed by PCR to verify the presence of binding products using NRF2 mRNA primer.

### 2.11 Evaluation of RNA stability

After transfection for 48 h, cells were treated with 10 μg/mL actinomycin D to terminate the RNA production in the cells. Then, qPCR was used to detect the residual quantity of the NRF2 mRNA at time points 0, 2, 4, 8 and 16 h, and half‐life periods of NRF2 mRNA in each group were calculated and compared.

### 2.12 Lipid peroxidation assay

After transfection for 48 h, cells were incubated with 1 mM Liperfluo (DojinDo) for 30 min at 37°C before they were harvested by trypsinization. Subsequently, cells were resuspended in fresh PBS strained through a 70 mM cutoff cell strainer and analysed using the 488 nm laser of a flow cytometer for excitation. Data were analysed using FlowJo Software.

### 2.13 ROS content detection

Cells were plated at 1 × 10^4^ density and seeded in culture flasks. ROS kit (S0033, Beyotime, Shanghai, China) was used to measure ROS levels according to the manufacturer's instructions.

### 2.14 Propidium iodide (PI) staining

Trypsin–EDTA (Invitrogen) was used to digest the cells to prepare a cell suspension and then the cell suspension was centrifuged at 1000 *g* for 3 min. The supernatant was removed, and the cell density was adjusted to 10^6^ cells/mL in PBS. Two hundred microlitres of cell suspension was transferred to a small test tube and 100 μL of PI staining solution was used to incubate the staining solution at 37°C for 15 min. The cells were observed with a fluorescence microscope with an excited wavelength of 545 nm to count dead cells (red). DAPI was used to label the nucleus (blue).

### 2.15 Determination of MDA and Fe^2+^ contents

The production of MDA in SW1990 cells was detected by a DetectX^®^ TBARS/MDA Universal Colorimetric Detection Kit (K077‐H, ARBOR ASSAYS, Ann Arbor, Michigan, USA) following the manufacturer's instructions. Similarly, the levels of Fe^2+^ were detected by Iron Colorimetric Assay Kits (Sigma, St. Louis, MO, USA).

### 2.16 Evaluation of mitochondrial DNA damage

Mitochondrial DNA damage was assayed with a mitochondrial DNA Damage Detection Kit (50‐753‐4394, Thermo Fisher Scientific) according to the manufacturer's instructions.

### 2.17 FerroOrange staining

FerroOrange (Fe^2+^ indicator, MX4559‐24UG, MKbio, Shanghai, China) was used to incubate the cells according to the manufacturer's instructions. The cells were observed with a fluorescence microscope with an excited wavelength of 532 nm.

### 2.18 Mitochondrial morphology observation by transmission electron microscope (TEM)

SW1990 cells (10^6^/well) were collected by centrifugation at 1000 rpm for 2 min. The cells were fixed with 2.5% glutaraldehyde at 4°C for 2.5 h and washed 5 times with phosphate buffer. Then, the cells were fixed with 1% osmium acid for 2 h, washed 3 times with phosphate buffer, fixed with 1% osmium tetroxide and dehydrated through an alcohol gradient until embedded in resin. The samples were detected using transmission electron microscopy (JEM‐1400 PLUS, Tokyo, Japan) after double staining with uranium acetate and lead citrate.

### 2.19 Histopathologic assessment

Tumour tissues were fixed in 4% buffered paraformaldehyde for 48 h and then embedded in paraffin, sectioned and stained with haematoxylin–eosin (H&E). For immunohistochemical (IHC) and immunofluorescence (IF) assays, briefly, tumour tissue sections were pretreated with trypsin (0.05%) for 10 min and then treated with 3% (vol /vol) H_2_O_2_. Sections were then blocked with 10% goat serum for 1 h at room temperature. After washing with PBS, anti‐HSPB1 antibody, anti‐FUS, or anti‐Ki67 (1:50 dilution) was applied to the sections, and the sections were incubated overnight at 4°C. Sections were then washed with PBS for 15 min and incubated with biotinylated secondary antibodies by using the Histostain Plus Kit (Invitrogen, Carlsbad, CA, USA). Sections were washed and incubated with 3,30‐diaminobenzidine (DAB) substrate for 2 min.

### 2.20 Statistical analysis

All statistical analyses were performed by using the SPSS software (ver. 21.0; SPSS, Chicago, IL). The quantitative data derived from three independent experiments were expressed as mean ± SD. Comparisons between two groups were made by the Student's *t*‐test. Data between multiple groups were performed with one‐way analysis of variance (ANOVA) followed by post hoc analysis with the Tukey test. *p* < 0.01 was considered statistically significant.

## 3 RESULTS

### 3.1 HSPB1 is screened as a potential regulator of ferroptosis in pancreatic cancer

**FIGURE 1:**
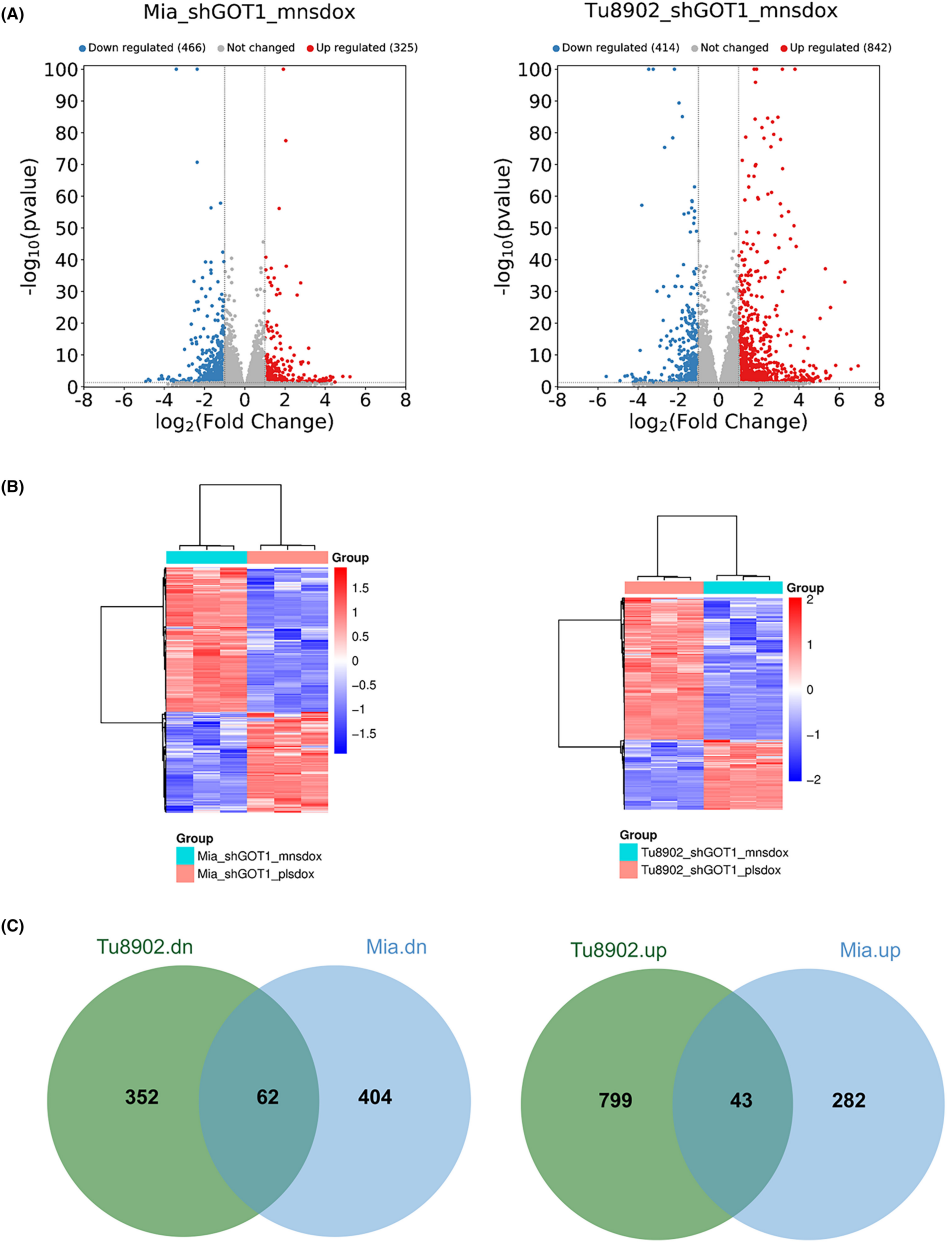
Bioinformatics analysis for the GSE157830 from GEO database. The GSE157830 experiment design: GOT1 was knocked down via doxycycline (Dox)‐inducible shRNA in two human pancreatic cancer cell lines, Tu8902 and MiaPaCa, and subject to RNA‐seq. For each cell line, three replicates each of control (‐Dox, “mnsdox”) and knockdown (+Dox, “plsdox”) were sequenced. High‐quality clean reads were aligned with the human reference genome (hg38) to get the raw gene level mRNA read counts. DESeq2 software (v1.30.1) was used to normalize and calculate the fold change and *p*‐value between two groups. Differentially expressed genes (DEGs) were screened for fold change ≥2 and *p* < 0.05. (A) Volcano maps for DEGs in GOT1 knocked down MiaPaCa (left panel) and Tu8902 (right panel) cells treated with or without Dox. (B) Heatmaps for DEGs in GOT1 knocked down MiaPaCa (left panel) and Tu8902 (right panel) cells treated with or without Dox. (C) The online bioinformatic tool Jvenn was used to intersect downregulated (left panel) and upregulated (right panel) DEGs between MiaPaCa and Tu8902 cells.

The aspartate aminotransaminase (GOT1) is a famous ferroptosis suppressor gene in pancreatic cancer. In the initial stage of our research, the GSE157830 dataset, containing expression profiles related to GOT1 knocked‐down human pancreatic cancer cell lines Tu8902 and MiaPaCa, was downloaded from the GEO database, and DEGs were analysed. The results showed that there were, respectively, 466 and 414 genes downregulated in response to doxycycline (Dox) treatment in GOT1 knocked‐down Tu8902 and MiaPaCa cells, and 325 and 842 genes upregulated, respectively (Figure 1A,B). The outputs of Jvenn showed that there were 62 genes both downregulated and 43 genes both upregulated in response to Dox treatment in GOT1 knocked‐down Tu8902 and MiaPaCa cells (Figure 1C).

**FIGURE 2:**
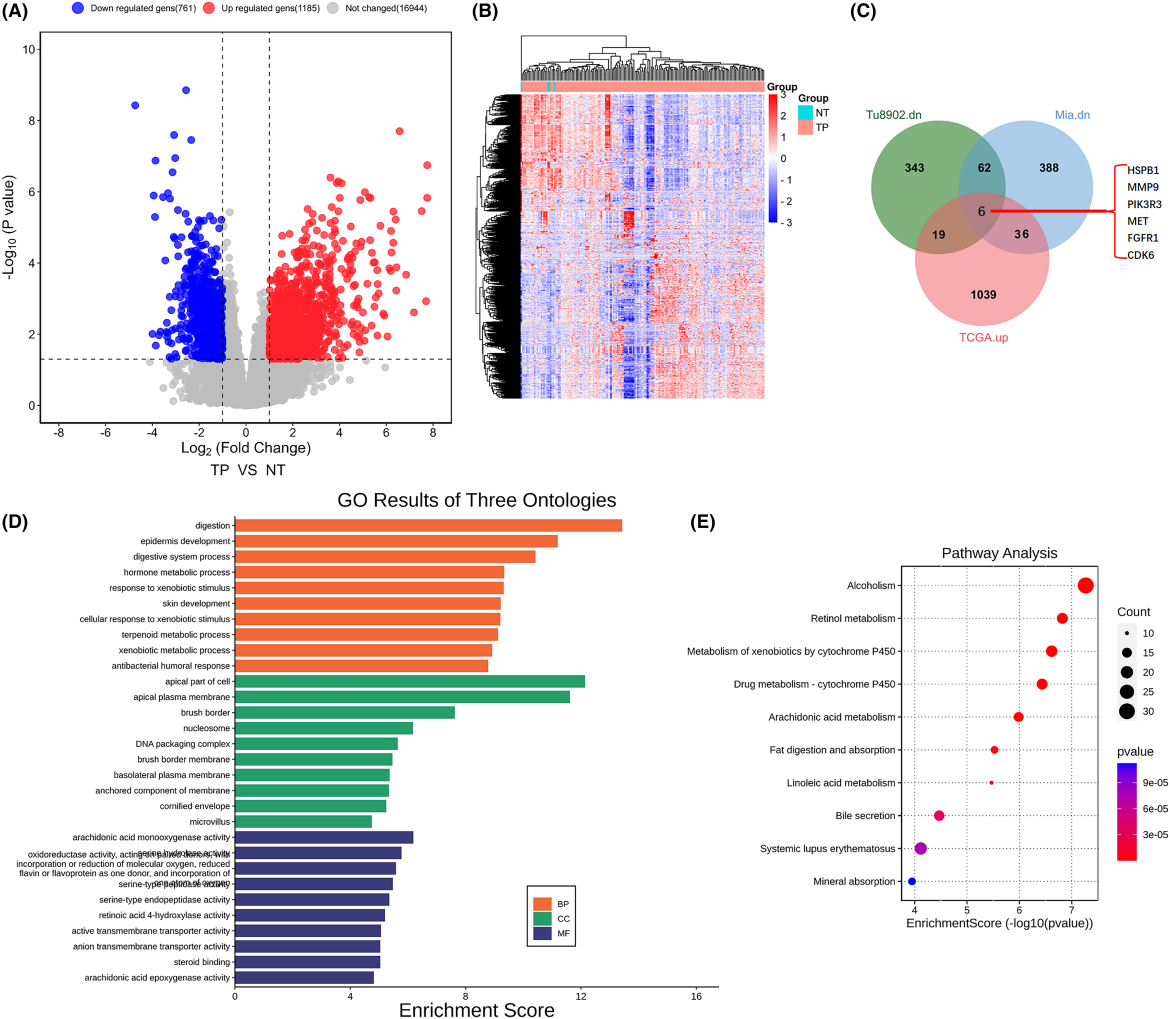
Combined bioinformatics analysis for GSE157830 dataset and gene expression matrix of the TCGA‐PAAD project. The gene expression matrix of the TCGA‐PAAD project was downloaded from GDC using R package TCGAbiolinks, and gene expression differential analysis was conducted using DESeq2, comparing TP (Primary Solid Tumour) with NT (Solid Tissue Normal). (A, B) Volcano maps and Heatmaps for DEGs between normal and PAAD tissues. (C) Jvenn was used to intersect the downregulated DEGs in Figure 1C and the upregulated DEGs of the PAAD project. (D, E) R package ClusterProfiler was used to perform GO and KEGG enrichment analyses of the intersection.

Then, the gene expression matrix of the TCGA‐PAAD (pancreatic adenocarcinoma) project was downloaded, and gene expression differential analysis was conducted. The volcanic map and heat map of DEGs showed that there are 761 downregulated and 1185 upregulated genes in PAAD tissues, respectively (Figure 2A,B). The online bioinformatic tool Jvenn was then used to intersect DEGs among the GSE157830 dataset and TCGA‐PAAD project, and the results showed that there were six intersecting genes that both downregulated in response to Dox treatment in GOT1 knocked‐down Tu8902 and MiaPaCa cells and simultaneously upregulated in PAAD tissues, including HSPB1, MMP9, PIK3R3, MET, FGFR1 and CDK6 (Figure 2C). Among these six genes, HSPB1 is an exosomal protein and a relatively less characterized regulator in pancreatic cancer. Box Plots from TCGA‐PAAD data showed that HSPB1 was significantly upregulated in PAAD tissues (Figure 2D). Moreover, GO and KEGG pathway enrichment analyses indicated that HSPB1 was associated with cell metabolism (Figure 2E), especially P450 monooxygenase metabolism, and enriched in pathways associated with monooxygenase activity (Figure 2F), suggesting a potential role of HSPB1 in the involvement of lipid peroxidation and ferroptosis.

### 3.2 HSPB1 is upregulated in human pancreatic cancer specimens and cell lines

**FIGURE 3:**
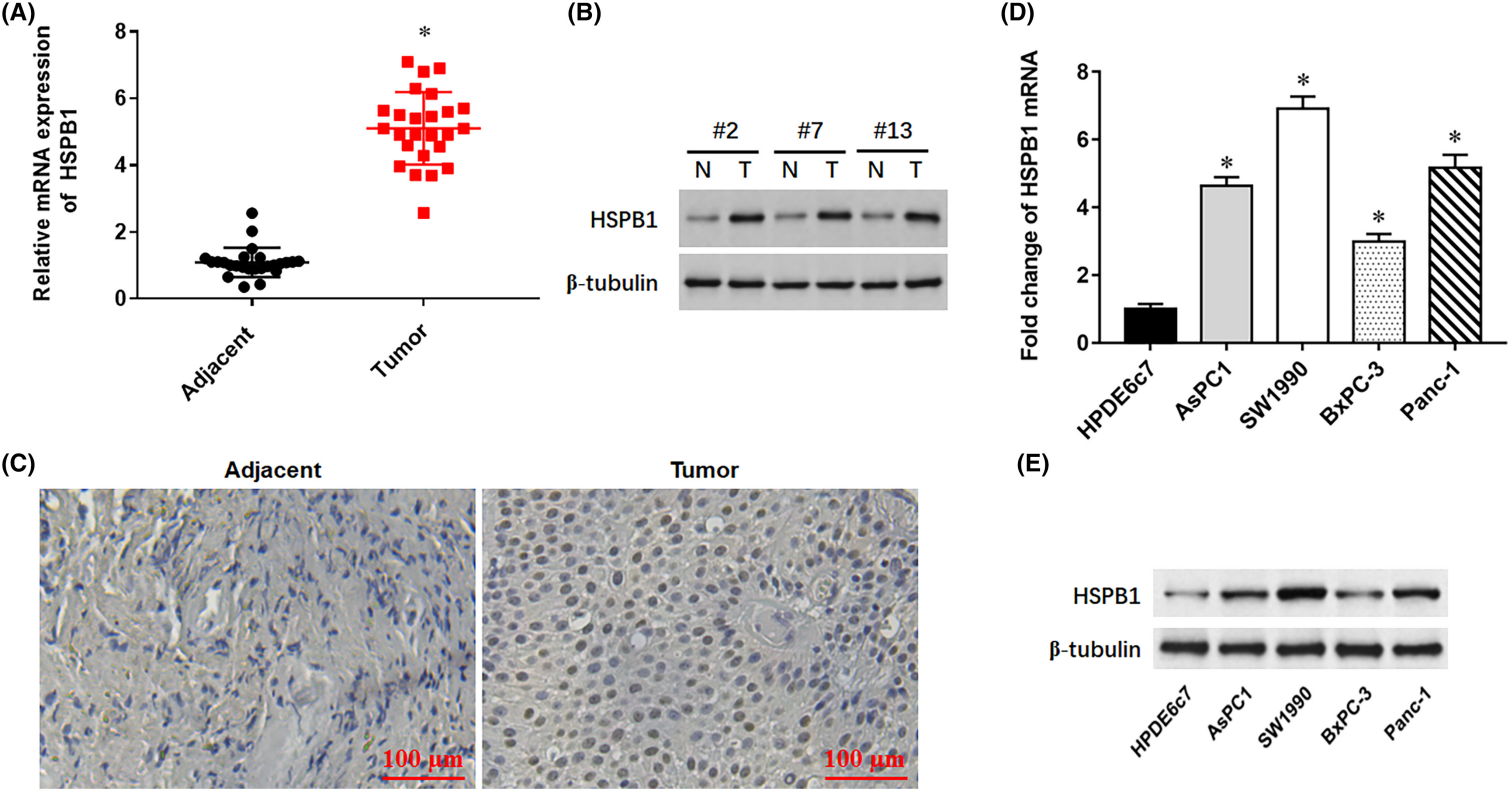
HSPB1 expression in human pancreatic cancer and cell lines. The pancreatic cancer tissues and matched adjacent tissues were collected from 30 patients. (A) RT‐qPCR was used to detect the expression levels of HSPB1 mRNA. **(**B) Western blotting was used to the expression levels of HSPB1 protein in 3 patients randomly selected. (C) Immunohistochemistry was used to detect HSPB1 expression in tumour tissues. (D, E) The levels of HSPB1 mRNA and protein were detected by qPCR and Western blot in pancreatic cancer cell lines AsPC1, SW1990, BxPC‐3 and Panc‐1, and the normal human pancreatic ductal epithelial cell line HPDE6c7. *p < 0.01.

Tumour tissues and matched paracancerous tissues were collected from 30 patients with pancreatic cancer, and RT‐qPCR results indicated that the expression of HSPB1 mRNA in tumour tissues was significantly higher than that in paracancerous tissues (Figure 3A). Representative Western blots and IHC images of the specimens showed that HSPB1 protein was also obviously upregulated in the tumour tissue and mainly located in the nucleus Furthermore, the expression of HSPB1 was upregulated in pancreatic cancer cell lines (Figure 3B,C). In the cell lines, HSPB1 mRNA and protein levels were significantly higher in AsPC1, SW1990, BxPC‐3 and Panc‐1, compared with normal human pancreatic ductal epithelial cell line HPDE6c7 (Figure 3D,E).

### 3.3 HSPB1 is upregulated in exosomes from human pancreatic cancer cells

**FIGURE 4:**
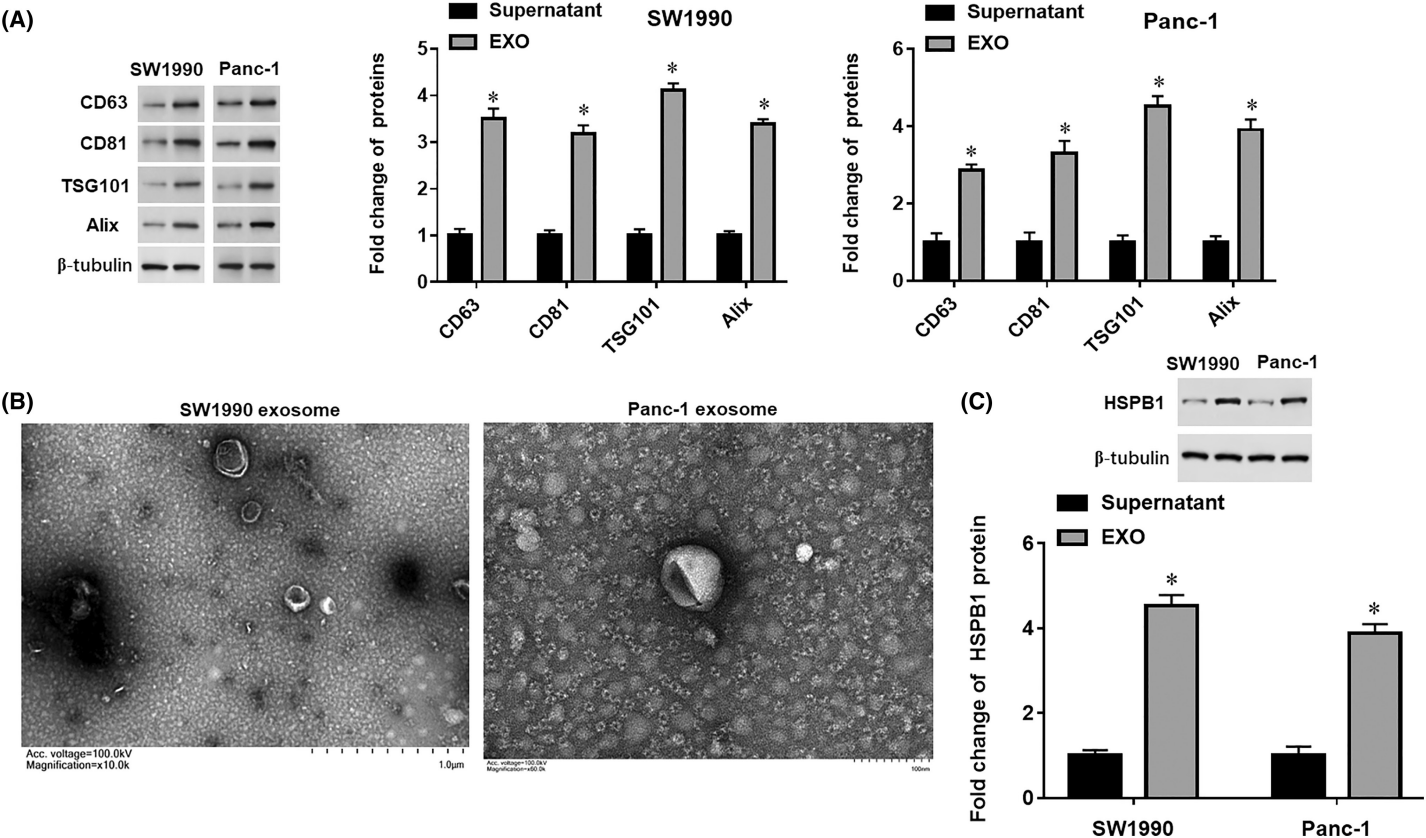
Isolation and identification of exosomes derived from human pancreatic cancer cells SW1990 and Panc‐1. The cell culture supernatant of SW1990 and Panc‐1 was, respectively, collected, and exosomes were isolated by ultracentrifugation. (A) The expression levels of exosome marker proteins in the supernatants and exosomes were detected by Western blotting. (B) Representative images of exosomes derived from SW1990 and Panc‐1 cells. (C) Western blotting was used to detect the protein expression of HSPB1 in exosomes. *p < 0.01.

Culture supernatants of human pancreatic cancer cells SW1990 and Panc‐1 were collected, and exosomes were extracted by ultracentrifugation. As expected, exosome marker proteins, including CD63, CD81, TSG101 and Alix, were enriched in exosomes but hardly detected in the cell supernatant with exosomes deprived (Figure 4A). Furthermore, the representative images of exosomes from human pancreatic cancer cells identified by TEM were shown in Figure 4B, which indicated that the extract exosomes had a characteristic morphology of round‐shaped particles. HSPB1 is a member of the heat shock protein family, which plays important roles under various stress conditions in cancerous and non‐cancerous tissues, such as hypoxia, ageing and hyperthermia.^9^, ^10^ Some studies revealed that HSPB1 is enriched in the exosomes derived from multiple cell types eroded by inflammation.^11^, ^12^, ^13^ However, it is not clear whether HSPB1 is enriched in gastrointestinal cancer cell‐derived exosomes and what is the exact role of exosome‐HSPB1 in pancreatic cancer. Next, Western blotting results confirmed that HSPB1 was abundantly enriched in exosomes derived from human pancreatic cancer cells (Figure 4C).

### 3.4 Exosomal HSPB1 enhances proliferation and invasion and suppresses ferroptosis in mildly hypoxic SW1990 cells

**FIGURE 5:**
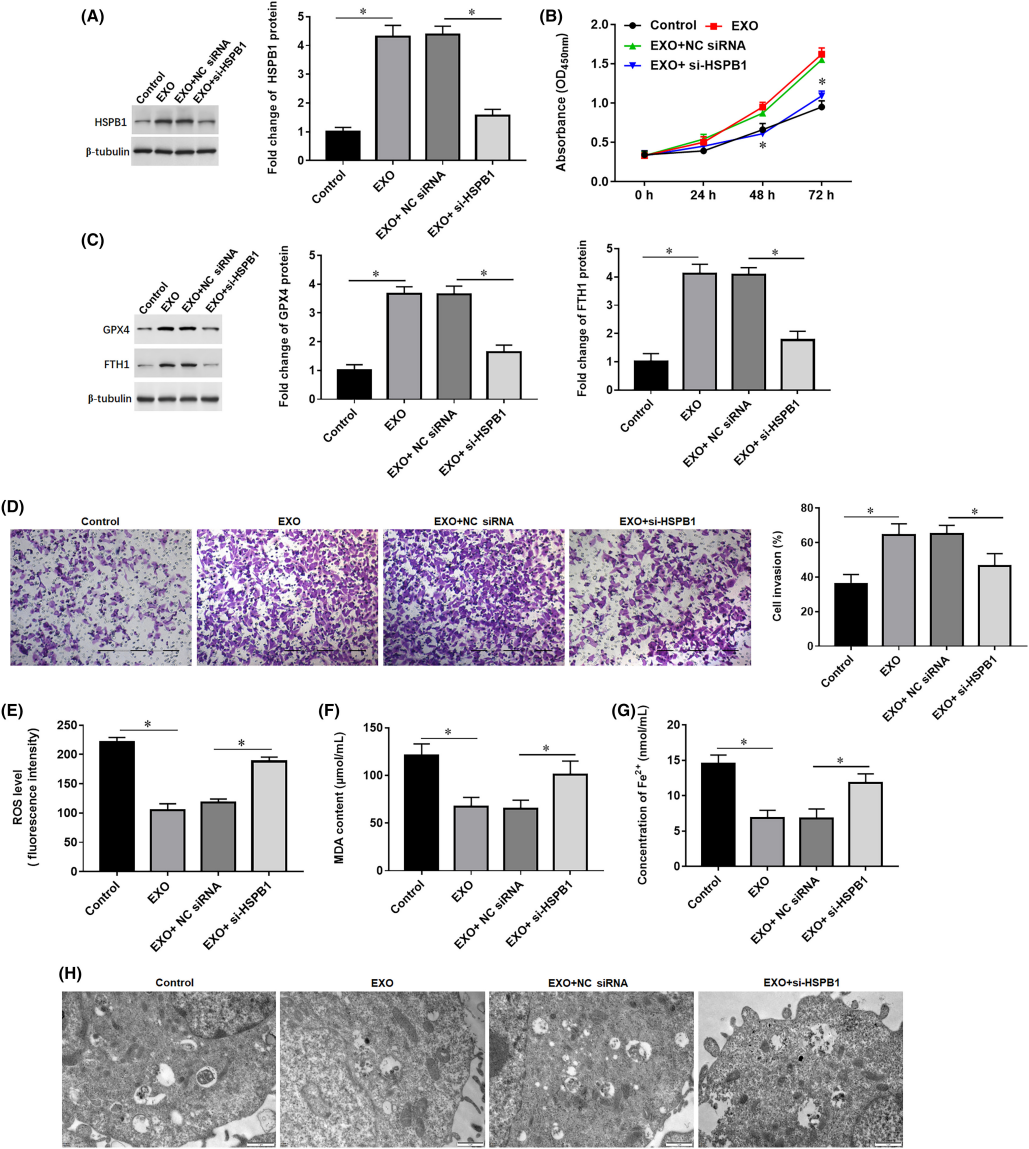
The effect of exosomal HSPB1 on SW1990 cell functions. Ten micrograms of exosomes was used to treat SW1990 cells alone or together with 60 nM HSPB1 siRNA (si‐HSPB1), and the cells were cultured under a mildly hypoxic condition (5% O_2_–5% CO_2_). After 48 h, the cells were harvested. (A) The protein expression of HSPB1 was detected by Western blotting. (B) Cell proliferation was analysed by CCK‐8 assay. (C) The invasion ability of cells was detected by the Transwell invasion assay (200×). (D) The protein expression of GPX4 and FTH1 in cells was detected by Western blotting. (E) Flow cytometry was used to detect the content of ROS. (F, G) The contents of MDA and Fe^2+^ were detected by using corresponding kits. (H) Observation of mitochondrial morphology with transmission electron microscopy. *p < 0.01.

Evidence indicates that mildly hypoxic condition (3%–10%) is an oxygen environment closer to the internal organs of the body and more suitable for targeted therapy and drug screening for tumour cells.^14^, ^15^ Exosomes were used to treat SW1990 cells alone or together with si‐HSPB1, and the cells were cultured under a mildly hypoxic condition (5% O_2_–5% CO_2_). We observed increased HSPB1 expression (Figure 5A) and enhanced proliferation (Figure 5B) and invasion (Figure 5C) in exosomes‐treated SW1990 cells, and transfection of si‐HSPB1 reversed the effect of exosomes (Figure 5A–C). PI staining showed that exosomes treatment significantly decreased the percentage of cell death (PI‐positive), while si‐HSPB1 reversed the inhibitory effect of exosomes on cell death (Supplementary Figure S1A). Western blotting confirmed that levels of anti‐ferroptosis marker proteins GPX4 and FTH1 were increased in exosomes‐treated cells (Figure 5D), which was also able to be reversed by si‐HSPB1. Next, we observed that exosomes treatment reduced ROS (Figure 5E) and MDA contents (Figure 5F), and decreased Fe^2+^ concentration (Figure 5G) and lipid peroxide levels (FerroOrange intensity, Supplementary Figure S1B) in SW1990 cells, and transfection of si‐HSPB1 reversed the effect of exosomes. Then, we observed the mitochondrial morphology with transmission electron microscopy. The results showed that mitochondria in mildly hypoxic SW1990 cells displayed slightly shrunken and thick‐ridged morphology, suggesting the occurrence of modest ferroptosis; mitochondrial morphology in EXO‐treated mildly hypoxic SW1990 cells looks nearly normal in size and morphology; while si‐HSPB1 reversed the improvement of exosomes on mitochondrial morphology (Figure 5H). Moreover, exosomes treatment significantly improved mitochondrial DNA (mtDNA) damage, while si‐HSPB1 reversed the improvement of exosomes on mtDNA damage (Supplementary Figure S1C). These data indicated that EXO‐treated suppresses hypoxia‐induced ferroptosis to promote proliferation and invasion in mildly hypoxic SW1990 cells, and knockdown of HSPB1 reversed the suppressive effect of EXO on ferroptosis.

### 3.5 HSPB1 interacts with and positively regulates the level of FUS protein

**FIGURE 6:**
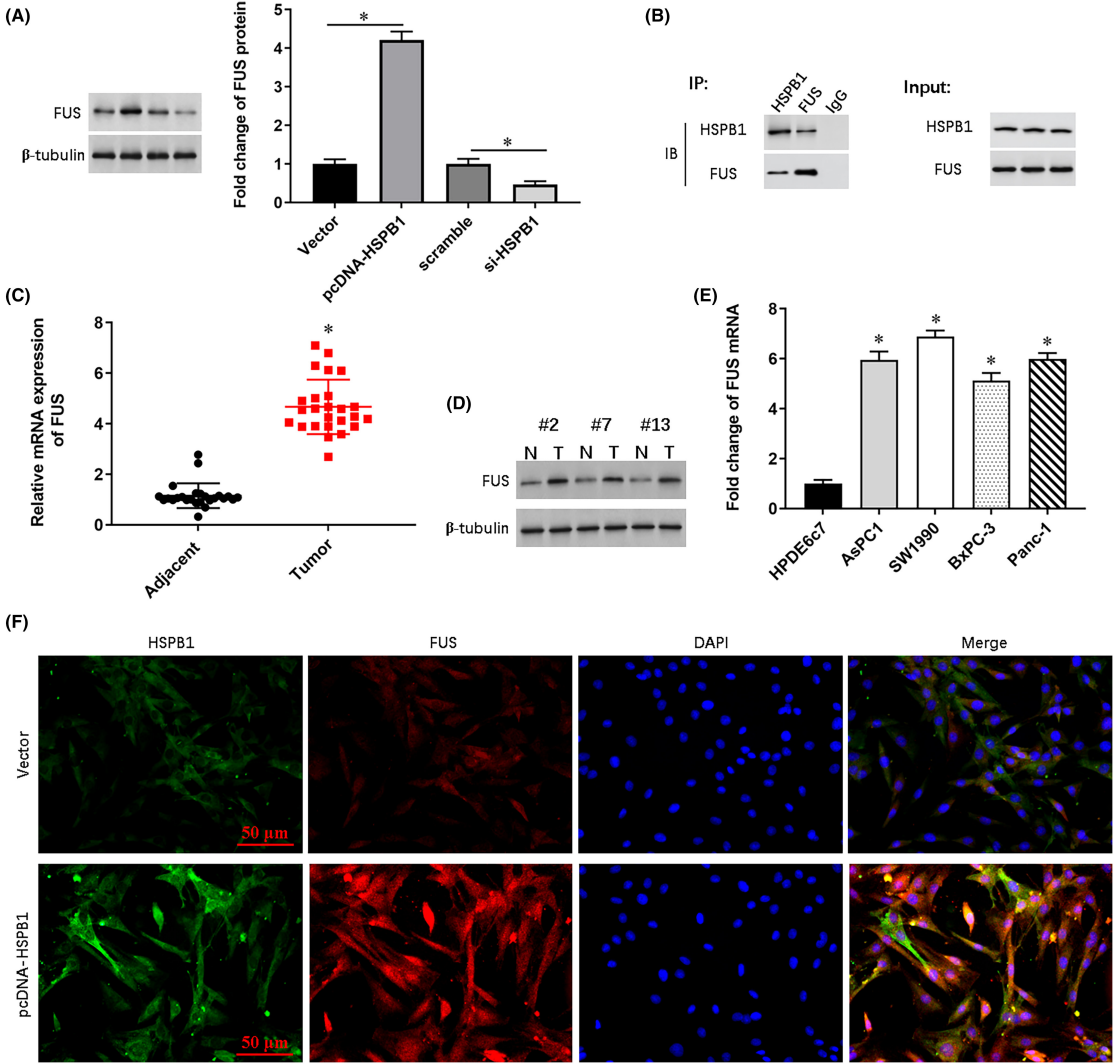
HSPB1 interacts with FUS. (A) The pcDNA‐HSPB1, si‐HSPB1 and their negative controls were, respectively, transfected into SW1990 cells, and the protein expression of FUS was detected by Western blotting. (B) Co‐immunoprecipitation assay was used to detect the binding of FUS and HSPB1. (C) RT‐qPCR was used to detect the expression levels of FUS mRNA in pancreatic cancer tissues and matched adjacent tissues. (D) Western blotting was used to the expression levels of FUS protein in three patients randomly selected. (E) The level of FUS mRNA was detected by qPCR in cell lines AsPC1, SW1990, BxPC‐3 and Panc‐1, and HPDE6c7. (F) Fluorescent antibodies of FUS and HSPB1 were used to label their expression in SW1990 cells transfected with vector or pcDNA‐HSPB1. *p < 0.01.

The results of online bioinformatics analysis indicated that FUS protein might be a potential target of HSPB1 (https://thebiogrid.org). Western blotting results showed that transfection of pcDNA‐HSPB1 promoted FUS protein expression, and transfection of si‐HSPB1 inhibited FUS expression (Figure 6A). The binding relationship between HSPB1 and FUS was verified by co‐immunoprecipitation (Figure 6B) using HSPB1 antibody and FUS antibody. RT‐qPCR and representative Western blots of the specimens showed that FUS mRNA and protein were upregulated in the tumour tissue (Figure 6C,D). In the cell lines, the FUS mRNA level was significantly higher in AsPC1, SW1990, BxPC‐3 and Panc‐1, compared with normal human pancreatic ductal epithelial cell line HPDE6c7 (Figure 6E). To confirm the interaction between HSPB1 and FUS, we used their fluorescent antibodies to label their expression in SW1990 cells transfected with vector or pcDNA‐HSPB1. The results showed that HSPB1 and FUS were co‐localized in cell nucleus and plasma, and HSPB1 overexpression had a positive effect on the expression of FUS protein (Figure 6F).

### 3.6 FUS is required for exosomal HSPB1‐induced suppression of lipid peroxidation and ferroptosis in mildly hypoxic SW1990 cells

**FIGURE 7:**
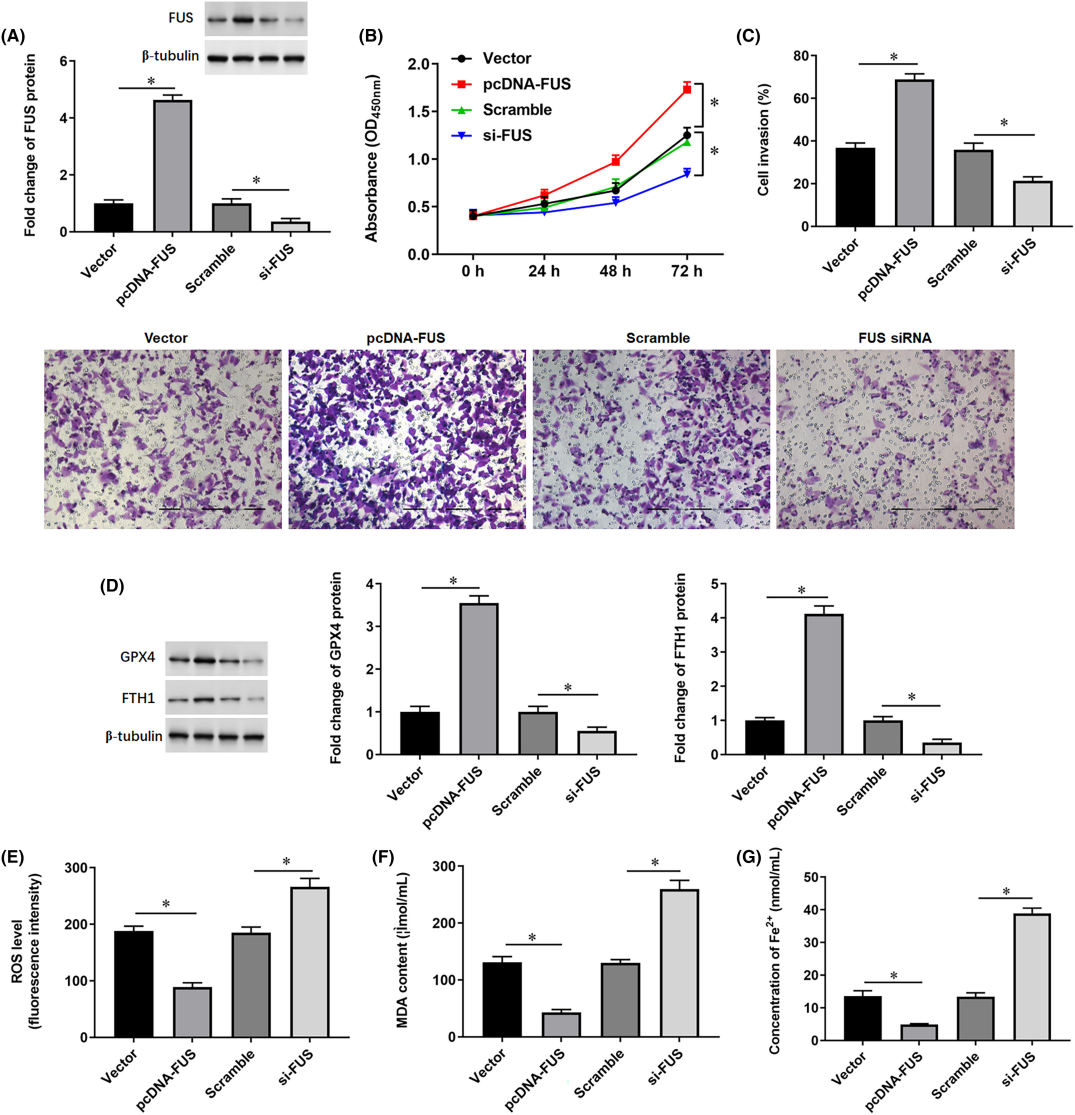
The effect of FUS on SW1990 cell functions. The pcDNA‐FUS or si‐FUS were transfected into SW1990 cells, respectively, and the cells were cultured in a mildly hypoxic condition. After transfection for 48 h, the cells were harvested. (A) The protein expression of FUS was detected by Western blotting. (B) Cell proliferation was analysed by CCK‐8 assay. (C) The invasion ability of cells was detected by the Transwell invasion assay (200×). (D) The protein levels of GPX4 and FTH1 were detected by Western blotting. (E) Flow cytometry was used to detect the content of ROS. (F, G) The contents of MDA and Fe^2+^ were detected by using corresponding kits. *p < 0.01.

To further investigate the role of FUS and its involvement in exosomal HSPB1‐mediated ferroptosis, the pcDNA‐FUS and si‐FUS were transfected into mildly hypoxic SW1990 cells, respectively. Western blotting showed that transfection of pcDNA‐FUS upregulated FUS expression and transfection of si‐FUS inhibited FUS expression (Figure 7A). Transfection of pcDNA‐FUS promoted cell proliferation (Figure 7B) and invasion (Figure 7C), increased the levels of anti‐ferroptosis proteins GPX4 and FTH1 (Figure 7E) and suppressed the production of ROS (Figure 7E), MDA (Figure 7F) and Fe^2+^ (Figure 7G). While transfection of si‐FUS had the opposite effect (Figure 7B–G).

**FIGURE 8:**
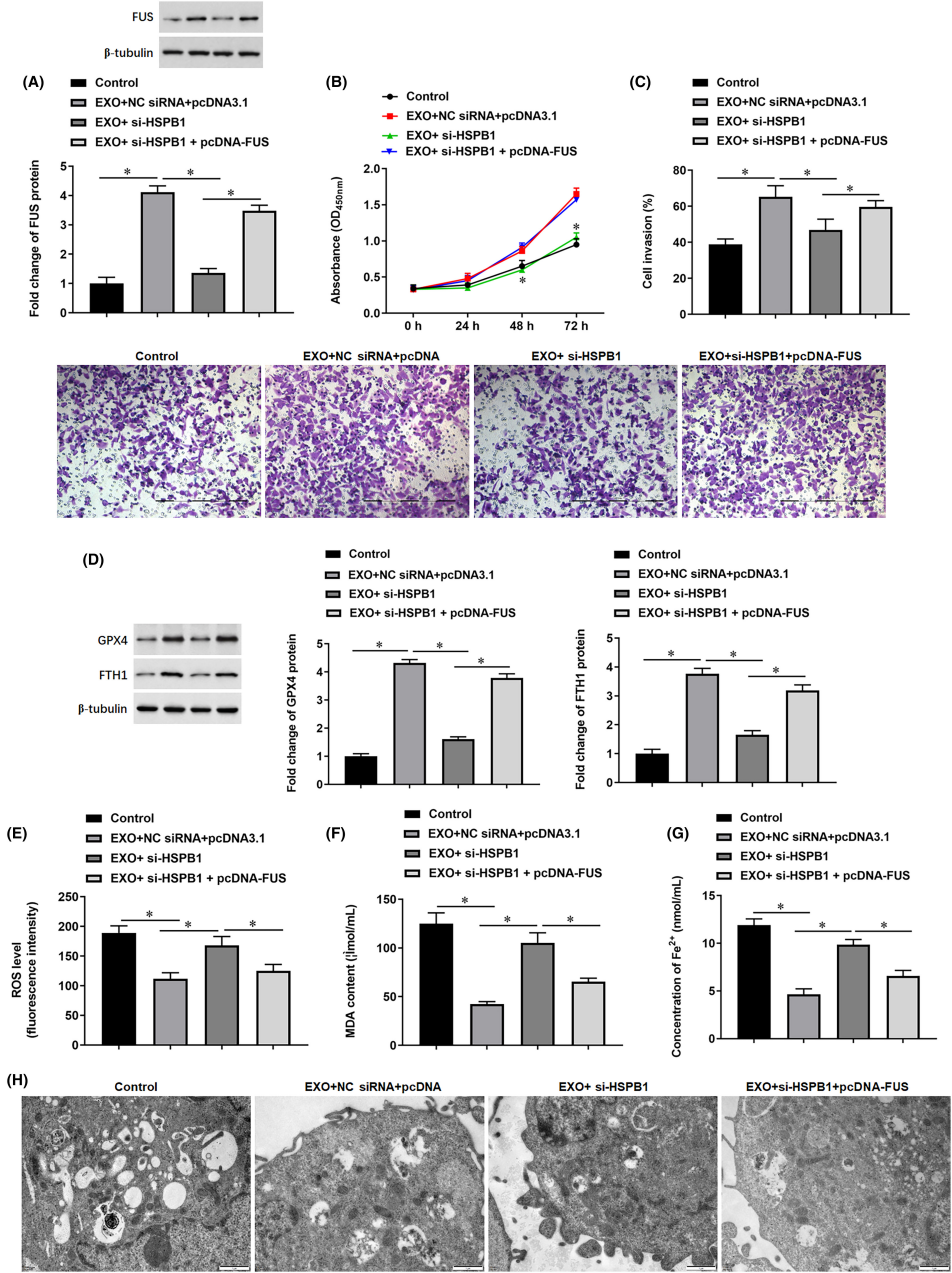
The involvement of FUS in exosomal‐mediated suppression of ferroptosis. Exosome‐incubated mildly hypoxic SW1990 cells were transfected with si‐HSPB1 or together with pcDNA‐FUS. (A) The protein level of FUS was detected by Western blotting. (B) Cell proliferation was analysed by CCK‐8 assay. (C) The invasion ability of cells was detected by the Transwell invasion assay. (D) The protein levels of GPX4 and FTH1 were detected by Western blotting. (E) Flow cytometry was used to detect the content of ROS. (F, G) The contents of MDA and Fe^2+^ were detected by using corresponding kits. (H) Observation of mitochondrial morphology with transmission electron microscopy. *p < 0.01.

Subsequently, exosome‐incubated mildly hypoxic SW1990 cells were transfected with si‐HSPB1 or together with pcDNA‐FUS. Our results showed that exosomes incubation promoted FUS protein expression (Figure 8A), enhanced cell proliferation (Figure 8B) and invasion (Figure 8C) and improved cell death (Supplementary Figure S2A); transfection of si‐HSPB1 decreased exosomes‐induced FUS protein expression, cell proliferation and invasion; while, transfection of pcDNA‐FUS reversed the effect of si‐HSPB1. In consistent with the changes in cell phenotypes, transfection of si‐HSPB1 decreased exosomes‐induced expression of anti‐ferroptosis proteins GPX4 and FTH1 (Figure 8D) and production of ROS (Figure 8E), MDA (Figure 8F), Fe^2+^ (Figure 8G) and lipid peroxide levels (Supplementary Figure S2B), and transfection of pcDNA‐FUS reversed the effect of si‐HSPB1 (Figure 8D–G). Moreover, transmission electron microscopy also showed that transfection of si‐HSPB1 reversed exosomes‐mediated improvement of mitochondrial morphology, while overexpression of FUS reversed the effect of HSPB1 knockdown (Figure 8H). Also, transfection of si‐HSPB1 reversed the improvement of exosomes on mtDNA damage, while overexpression of FUS rescued the effect of HSPB1 knockdown (Supplementary Figure S2C). These findings indicated that FUS is required for exosomal HSPB1‐induced suppression of ferroptosis in mildly hypoxic SW1990 cells.

To further confirm the role of HSPB1 in ferroptosis, normoxic SW1990 cells were treated with the ferroptosis inducer Erastin or together with exosomes. Compared with mild hypoxia, Erastin treatment induced a modestly stronger decrease in the expression levels of HSPB1, FUS, GPX4 and FTH1 proteins (Supplementary Figure S3A). Consistently, Erastin treatment induced a modestly stronger increase in the production of ROS (Supplementary Figure S3B), MDA (Supplementary Figure S3C), Fe^2+^ (Supplementary Figure S3D), and mtDNA damage (Supplementary Figure S3E), and induced a modestly stronger decrease in cell proliferation and invasion (Supplementary Figure S3F,G). Exosomes treatment was able to almost completely reversed Erastin‐induced ferroptosis and suppression of cell proliferation and invasion.

### 3.7 Activation of Nrf2/HO‐1 pathway is required for exosomal HSPB1‐induced suppression of lipid peroxidation and ferroptosis

**FIGURE 9:**
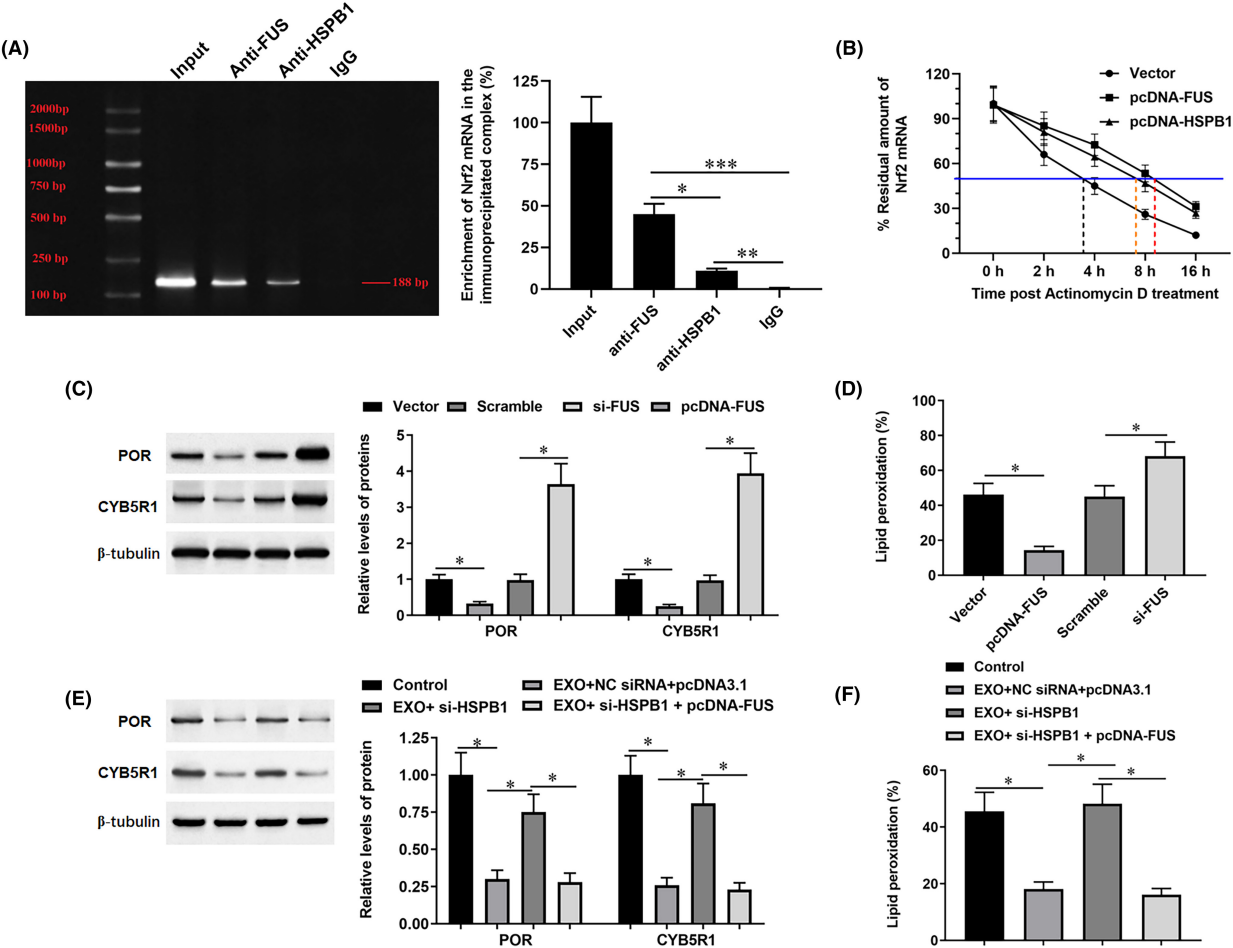
The regulation of FUS on NRF2 and P450 monooxygenase. (A) RNA‐IP was used to verify the binding of FUS protein to NRF2 mRNA. (B) The pcDNA‐HSPB1 and pcDNA‐FUS were used to transfect SW1990 cells, and after 48 h, Actinomycin D was applied to treat the cells and residual amounts of NRF2 mRNA were detected to evaluate its stability. Then, the pcDNA‐FUS or si‐FUS were transfected into mildly hypoxic SW1990 cells, and the protein levels of POR and CYB5R1 (C) and the level of lipid peroxidation (D) were detected by Western blotting. Subsequently, exosome‐incubated mildly hypoxic SW1990 cells were transfected with si‐HSPB1 or together with pcDNA‐FUS, and the protein levels of POR and CYB5R1 (E) and the level of lipid peroxidation (F) were detected. *p < 0.01.

A previous study revealed that, as a famous RNA‐binding protein, FUS was able to bind to and stabilize the NRF2 mRNA.^16^ Here, we verify the regulation of FUS protein on NRF2 expression in SW1990 cells. Our RNA‐IP results showed that FUS protein is directly bound to NRF2 mRNA (Figure 9A). RNA stability assay using Actinomycin D showed that overexpression of HSPB1 and FUS both notably extended the decay of NRF2 mRNA (Figure 9B). Furthermore, FUS negatively regulated the expression of P450 isoforms POR and CYB5R1 and lipid peroxidation (Figure 9C,D) in mildly hypoxic SW1990 cells. Consistently, exosomes‐treated suppressed expression of POR and CYB5R1 and lipid peroxidation, transfection of si‐HSPB1 reversed the effect of exosomes, while overexpression FUS reversed the effect of si‐HSPB1 (Figure 9E,F).

**FIGURE 10:**
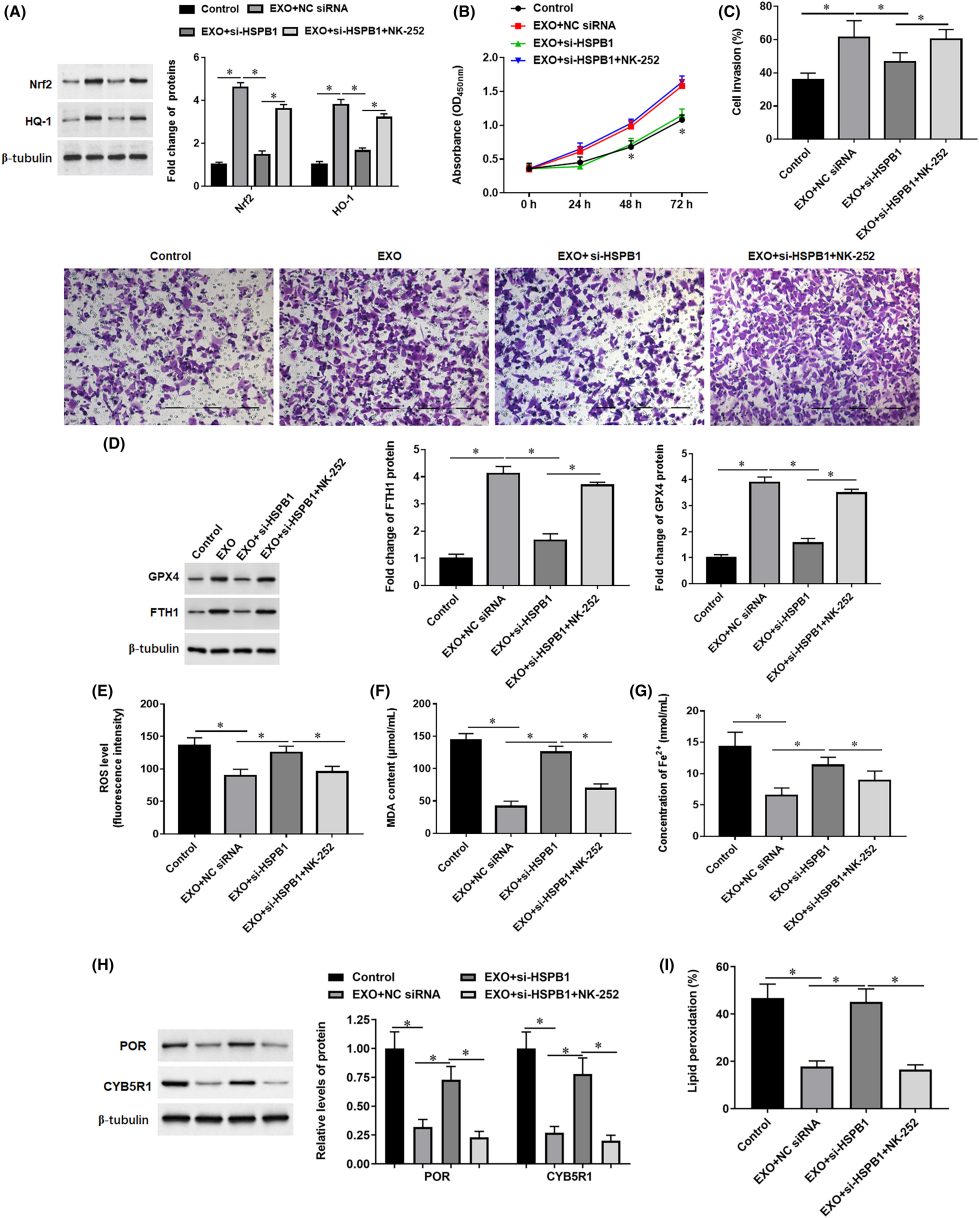
Activation of Nrf2/HO‐1 reversed the effect of si‐HSPB1 in Exosome‐incubated mildly hypoxic SW1990 cells. Exosome‐incubated mildly hypoxic SW1990 cells were transfected with si‐HSPB1 alone or incubated together with NK‐252, an Nrf2 activator. (A) The protein expression of Nrf2 and HO‐1 in cells was detected by Western blotting. (B) Cell proliferation was analysed by CCK‐8 assay. (C) The invasion ability of cells was detected by the Transwell invasion assay (200×). (D) The protein levels of GPX4 and FTH1 were detected by Western blotting. (E) Flow cytometry was used to detect the content of ROS. (F, G) The contents of MDA and Fe^2+^ were detected by using corresponding kits. The protein levels of POR and CYB5R1 (H) and the level of lipid peroxidation (I) were detected. *p < 0.01.

Exosome‐incubated SW1990 cells were transfected with si‐HSPB1 alone or incubated together with NK‐252, a Nrf2 activator. Western blotting results showed that the protein expression of Nrf2 and HO‐1 (Figure 10A) was increased after being treated with exosomes, transfection of si‐HSPB1 decreased Nrf2 and HO‐1 protein expression, and this effect was abrogated by NK‐252. Moreover, transfection of si‐HSPB1 suppressed exosomes‐induced cell proliferation (Figure 10B) and invasion (Figure 10C), expression of GPX4 and FTH1 (Figure 10D), suppression of production of ROS (Figure 10E), MDA (Figure 10F), Fe^2+^ (Figure 10G), P450 expression (Figure 10H) and lipid peroxidation (Figure 10I). Whereas, the effect of si‐HSPB1 was abrogated by NK‐252, an activator of NRF2/HO‐1 signalling (Figure 10B–I). These data indicated that activation of the Nrf2/HO‐1 pathway is required for exosomal HSPB1‐induced suppression of lipid peroxidation and ferroptosis.

### 3.8 Knockdown of HSPB1 inhibits tumour growth and NRF2/HO‐1/P450 anti‐ferroptosis signalling in vivo

**FIGURE 11:**
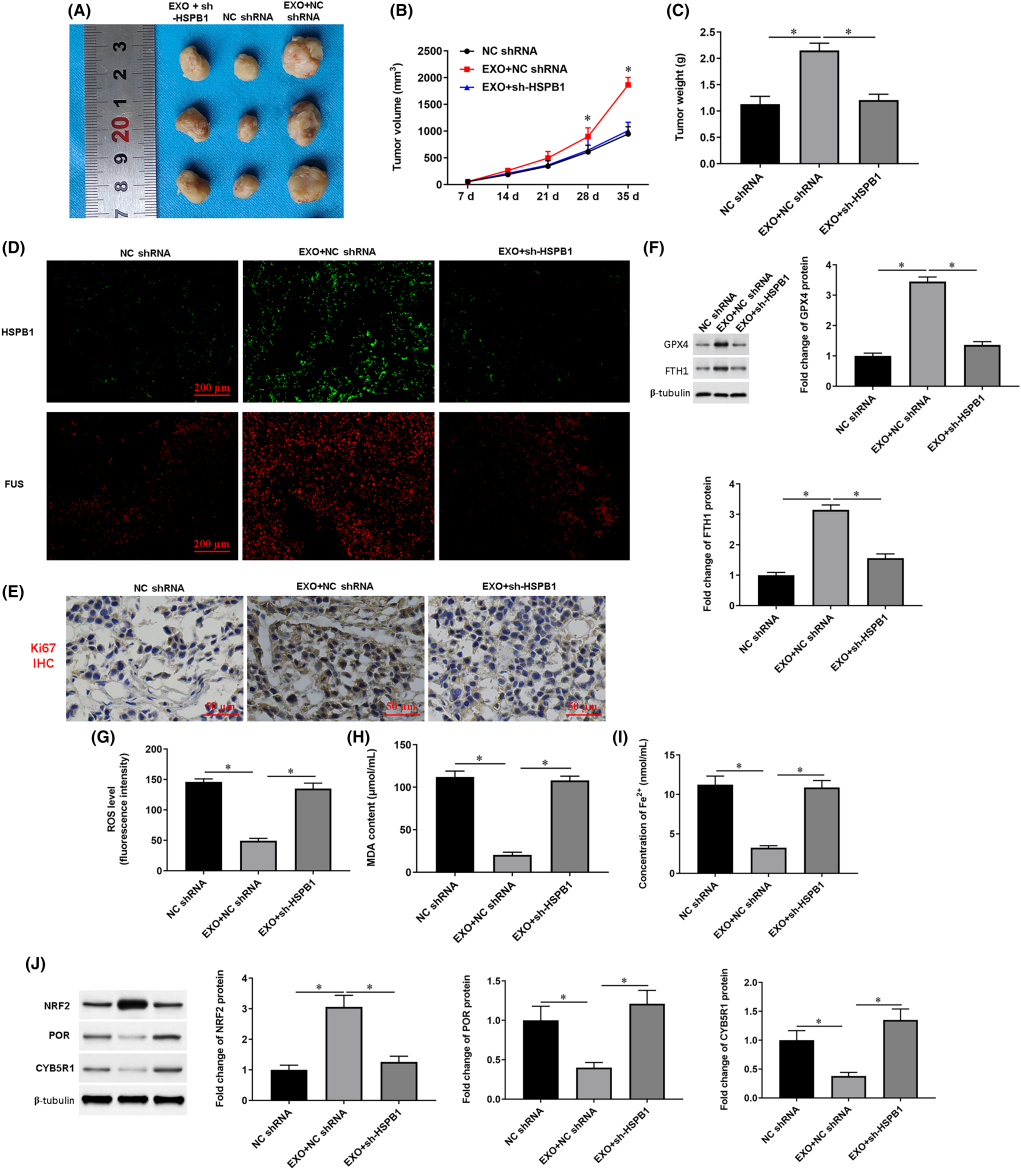
Knockdown of HSPB1 expression inhibits tumour progression in vivo. A total of 10^7^ SW1990 cells were subcutaneously injected into the armpit of nude mice to establish a xenotransplantation model of human pancreatic cancer. Xenotransplantation nude mice were randomly divided into three groups (*n* = 8 per group) including Scramble group (locally injected with lentiviral interference vector inserted with scrambled shRNA, weekly, 200 μL, 10^13^ PFU/mL), EXO group (locally injected with scrambled shRNA plus exosomes, weekly, 200 μL), and EXO+sh‐HSPB1 group (locally injected with exosomes plus scrambled shRNA, weekly, 200 μL, respectively). (A) Representative tumour images at day 35 post‐injection. (B) Growth curve of the tumours based on the tumour volume. (C) Tumour weights. (D) The expression of HSPB1 and FUS proteins was evaluated by immunofluorescence assay in tumour tissues. (E) Immunohistochemistry was used to detect Ki67 protein expression in tumour tissues. (F) The protein levels of GPX4 and FTH1 in tumour tissues were detected by Western blotting. (H) Flow cytometry was used to detect the content of ROS. (G, I) The contents of MDA and Fe^2+^ were detected by using corresponding kits. (J) The protein levels of POR and CYB5R1 were detected with Western blotting. *p < 0.01.

Finally, xenotransplantation of human pancreatic cancer was established by injection with SW1990 cells in nude mice. The xenotransplant mice were administrated with a lentiviral interference vector inserted with scrambled shRNA, EXO+ scrambled shRNA, and EXO+HSPB1 shRNA. The representative tumour images are shown in Figure 11A. we observed that the tumour volume and weight were greater in nude mice injected with exosomes, and the knockdown of HSPB1 induced a decrease in the tumour volume (Figure 11B) and weight (Figure 11C). Immunofluorescence and immunohistochemistry assays showed that exosomes promoted the expression of HSPB1, FUS and ki‐67 in tumour tissues, and knockdown of HSPB1 reversed the effects of exosomes (Figure 11D,E). Moreover, the protein levels of GPX4, FTH1 and NRF2 (Figure 11F,J) were induced and levels of ROS, MDA, Fe^2+^ and lipid peroxidation were suppressed by exosome administration (Figure 11G–J), which was reversed by knockdown of HSPB1. These data indicated that knockdown of HSPB1 inhibits tumour growth and NRF2/HO‐1/P450 anti‐ferroptosis signalling in vivo.

**FIGURE 12:**
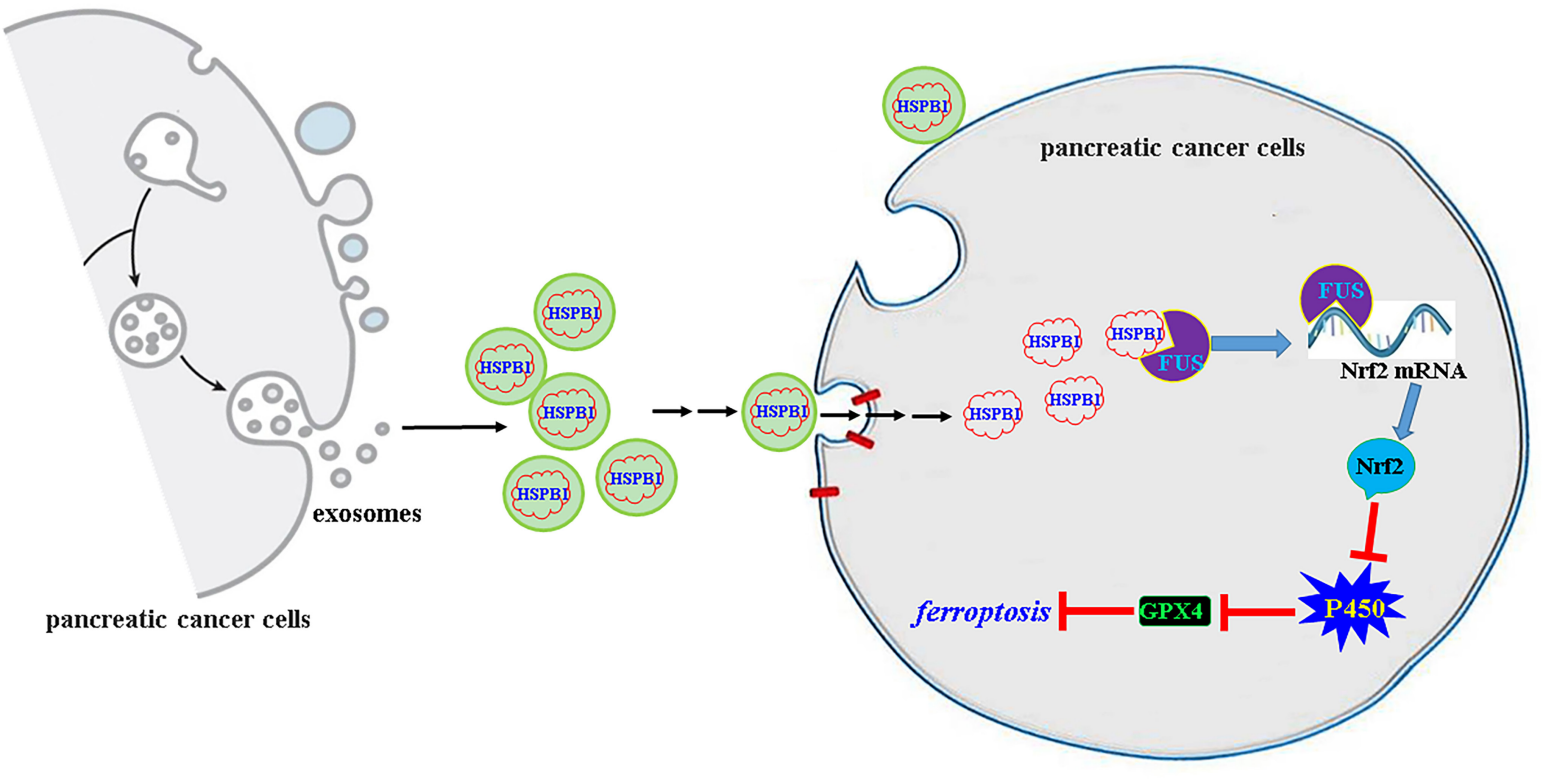
A diagrammatic sketch for the role and mechanism of exosomal HSPB1 in mildly hypoxic pancreatic cancer cells.

In summary, pancreatic cancer cells secret exosomes containing abundant HSPB1 protein. Exosomes enter surrounding pancreatic cancer cells and release the exosomal HSPB1 protein. HSPB1 interacts with the RNA binding protein FUS, which can bind and stabilize the NRF2 mRNA, activates the NRF2/HO‐1 anti‐ferroptosis signalling, suppresses P450‐mediated lipid peroxidation and regulates GPX4 expression, thus inhibiting ferroptosis and promoting cell survival and invasion (Figure 12).

## 4 DISCUSSION

Research over the past decade or so has shown that the occurrence and development of tumours are not simply manipulated by the alteration of oncogenes or tumour suppressor genes in the tumour cells themselves, but the tumour microenvironment also plays an important role in the malignant transformation of tumours. In this study, we screened HSPB1 as an exosomal protein regulator for ferroptosis of pancreatic cancer cells, and pancreatic cancer cell‐derived exosomal HSPB1 played a promoting role in pancreatic cancer progression by interacting with the RNA binding protein FUS and suppressing FUS/NRF2/HO‐1/P450‐mediated ferroptosis.

Ferroptosis is an iron‐dependent type of programmed cell death, which is emerging as a key mechanism involved in various major human diseases including pancreatic cancer. Apart from leading to changes in cell death, ferroptosis can affect many other functions of cancer cells, including migration/metastasis, invasion, drug resistance, metabolism and immune escape. Inhibition of ferroptosis has been demonstrated to be associated with increased migration, invasion and metastasis of multiple cancer cells, such as renal cell carcinoma, gastric cancer and osteosarcoma.^17^, ^18^, ^19^, ^20^ In pancreatic cancer, inhibition of ferroptosis has also been regarded as an effective strategy to suppress cell migration and invasion. For example, solasonine treatment disrupted glutathione metabolism and induced SLC7A11‐mediated ferroptosis to block tumour formation and metastasis in mouse xenograft models.^21^ The lncRNA A2M‐AS1 and small‐molecule MMRi62 were demonstrated to inhibit cellular proliferation, migration, and invasion as well as the tumour growth of pancreatic cancer.^22^, ^23^ Ferroptosis is a complex metabolic disorder process, which includes iron overload, lipid peroxidation, and dysfunction of cellular antioxidant systems. Current thinking holds that ferroptosis shares multiple initiators and overlapping pathways with epithelial‐mesenchymal transition (EMT) which is a key foundation for tumour cell metastasis.^24^ Evidence also indicates that ferroptosis plays a critical role in angiogenesis that provides convenience for tumour cell metastasis.^25^

Exosomes are important media for communication between tumour cells and also an important component of the tumour microenvironment. It was reported that human pancreatic cancer cells SW1990 derived exosomes promoted the proliferation, migration and cell cycle progression of HPDE cells, while exosomes with downregulated expression of hsa_circ_0000069 inhibited HPDE cell proliferation, migration and cell cycle progression by inhibiting STIL expression and inhibited pancreatic cancer progression.^26^ Other studies demonstrated that exosomes from gemcitabine‐resistant pancreatic cancer stem cells mediate the horizontal transfer of drug‐resistant traits to gemcitabine‐sensitive pancreatic cancer cells by delivering miR‐210^27^ or miR‐520‐5p.^28^ In this study, we reported that SW1990 cells‐derived exosomes delivered HSPB1 protein into surrounding cells, and HSPB1 interacted with the RNA binding protein FUS functioning as a proto‐oncogene.

HSPB1, also known as HSP27, is mapped to chromosome 7q11 23, includes 2 introns and 3 exons, consists of 132–186 amino acids and has a molecular weight of 27 KD. The N‐terminus is the structural basis of HSP27, to which some molecular signature proteins can bind, and is composed of sequence relatively conserved phenylalanine and proline residues, while the C‐terminus harbours the crystallin domain consisting of 80–100 highly conserved amino acid residues, common to all tiny heat shock protein family members.^9^ As an important member of the HSP family, HSPB1 plays important roles under various stress conditions in cancerous and non‐cancerous tissues, such as hypoxia, ageing and hyperthermia.^9^, ^10^ For example, in hypoxic–ischemic (HI) rat brain, overexpression reduced ferritin levels, attenuated apoptosis, and augmented GPX4 and SLC7A11 levels in the hippocampus tissues, playing a neuroprotective role.^29^ Recently, some studies revealed that HSPB1 is enriched in the exosomes derived from multiple cell types associated with or eroded by inflammation, such as cerebral astrocytes, monocytes/macrophages and neuroblastoma cells.^11^, ^12^, ^13^ However, it is not clear whether HSPB1 is enriched in gastrointestinal cancer cell‐derived exosomes and what is the exact role of exosome‐HSPB1 in pancreatic cancer. In this study, we reported that HSPB1 is enriched in exosomes‐derived pancreatic cancer cells and functions as a proto‐oncogene by suppressing Nrf2/P450‐mediated ferroptosis.

A previous study showed that HSPB1 was abnormally highly expressed in pancreatic cancer tissues and promoted pancreatic cancer progression.^30^ In this study, we found that HSPB1 was enriched in pancreatic cancer cell‐derived exosomes and played a promoting role in pancreatic cancer by suppressing ferroptotic cell death. Similar to our study, a couple of studies have also revealed that HSPB1 played a suppressive role in cell ferroptosis in some other cell types. HSPB1 was demonstrated to inhibit ferroptosis through multiple mechanisms, such as activating P38 MAPK signalling, interacting and promoting G6PD expression, and modulating p53 signalling. In solid cancer tissues, the HSPB1/p38 MAPK and HSPB1/ERK1/2 (p42/p44 MAPKs) pathways were activated, and activation of the HSPB1/MAPKs conferred tumour cells with resistance to ferroptosis and contributed to cancer progression.^31^, ^32^ In the hypoxic–ischemic brain, HSPB1 was also reported to negatively regulate ferroptosis in hippocampal neurons by interacting and promoting G6PD expression.^29^ Our study firstly reported the role of the suppressive role of HSPB1 in hypoxic pancreatic cancer cells and also proposed a novel mechanism of HSPB1 in regulating ferroptosis. HSPB1 is also famous as a key regulator of p53, which interacts with p53 and mutually influences each other's expression and downstream pathways.^33^ Li et al. reported that MMRi62, a small molecule targeting the MDM2‐MDM4 axis, was able to induce degradation of FTH1 and mutant p53 to inhibit proliferation, clonogenic, and spheroid growth in pancreatic cancer cell lines harbouring either KRAS and TP53 double mutations or single TP53 mutation by inducing ferroptosis.^23^ HSPB1 is most famous for its involvement in regulating the stability and expression of p53, a classic inhibitor gene of apoptosis and also an inhibitor gene of ferroptosis in tumour cells.^34^, ^35^ Therefore, targeting HSPB1 may be a promising strategy to modulate ferroptosis of pancreatic cancer cells via multiple pathways.

FUS was frequently involved in the pathogenesis of multiple cancers and many other server diseases for its abundant regulatory functions and mechanisms.^36^ A few recent studies also revealed that FUS played a promoting role in the progression of pancreatic cancer by regulating cell cycle‐associated factors CCND1 and p27.^37^, ^38^ As a well‐characterized RNA binding protein, FUS was able to bind to the mRNA of many important genes and play important roles based on its target genes. For example, FUS bound to the mRNA of EZH2, the famous core protein of the polycomb repressive complex 2 (PRC2), stabilized the EZH2 mRNA and regulated EZH2‐mediated suppression of PTEN to promote proliferation, migration and invasion of laryngeal squamous cell cancer cells.^39^ In this study, we demonstrated that FUS bound to and stabilized the mRNA of the ferroptosis suppressor gene NRF2 and regulated NRF2‐mediated suppression of ferroptosis, thus contributing to pancreatic cancer cell proliferation and invasion.

Nrf2/HO‐1 is one of the major anti‐oxidative signalling pathways during cell metabolism, which could be activated by multiple upstream regulators and function as a sweeper lipid peroxidation in stressed cells.^40^ As known, in non‐malignant hypoxic or ischemic tissues, Nrf2/HO‐1 signalling was depressed by overload of superoxide anions/hydroxyl radical/peroxynitrite and free radicals of lipids that were catalysed by oxidoreductases, such as P450s, and restore of the activation of Nrf2/HO‐1 signalling protected tissue cells against oxidative stress and inflammatory damage.^41^, ^42^, ^43^ Ferroptosis is a type of cell death caused by a redox imbalance between the production of oxidants and antioxidants, driven by the multiple redox‐active enzymes.^44^ Except for overloaded Fe concentration, excessive lipid peroxidation is an outstanding feature of ferroptosis. Accordingly, Nrf2/HO‐1/GPX4 was regarded as one of the most concerned anti‐ferroptosis pathways in tumours and non‐cancerous tissues.^45^, ^46^, ^47^ In this study, we reported that Nrf2/HO‐1 was activated by cancer cell‐derived exosomal HSPB1 in hypoxic pancreatic cancer cells via FUS protein, and knockdown of HSPB1 or FUS caused suppression of Nrf2/HO‐1, increased the level of the ferroptosis fuel P450 oxidases,^48^, ^49^ and inhibited pancreatic cancer progression in vitro and in vivo.

In conclusion, we screened HSPB1 as an exosomal protein regulator for ferroptosis of pancreatic cancer cells, and exosomal HSPB1 played a promoting role in pancreatic cancer progression by interacting with the RNA binding protein FUS and suppressing FUS/NRF2/HO‐1/P450‐mediated ferroptosis. Our findings may provide a potential therapeutic target for pancreatic cancer.

## AUTHOR CONTRIBUTIONS

**Lun Zhang:** Conceptualization (equal); writing – original draft (equal); writing – review and editing (equal). **Liuxu Yang:** Data curation (equal); validation (equal); writing – review and editing (equal). **Keyuan Du:** Data curation (equal); validation (equal); visualization (equal); writing – review and editing (equal).

## CONFLICT OF INTEREST STATEMENT

The authors confirm that there are no conflicts of interest.

## Supporting information


Supplementary Figure S1.

## Data Availability

The datasets used during the present study are available from the corresponding author upon reasonable request.
