# Supplementary material for: Exosomal HSPB1, interacting with FUS protein, suppresses hypoxia‐induced ferroptosis in pancreatic cancer by stabilizing Nrf2 mRNA and repressing P450
Source: J Cell Mol Med. 2024 Apr 29;28(9):e18209. doi: 10.1111/jcmm.18209 (PMC11056849; doi:10.1111/jcmm.18209)
Supplement: Supplementary file 1 — Supplementary Figure S1. [file JCMM-28-e18209-s001.docx]

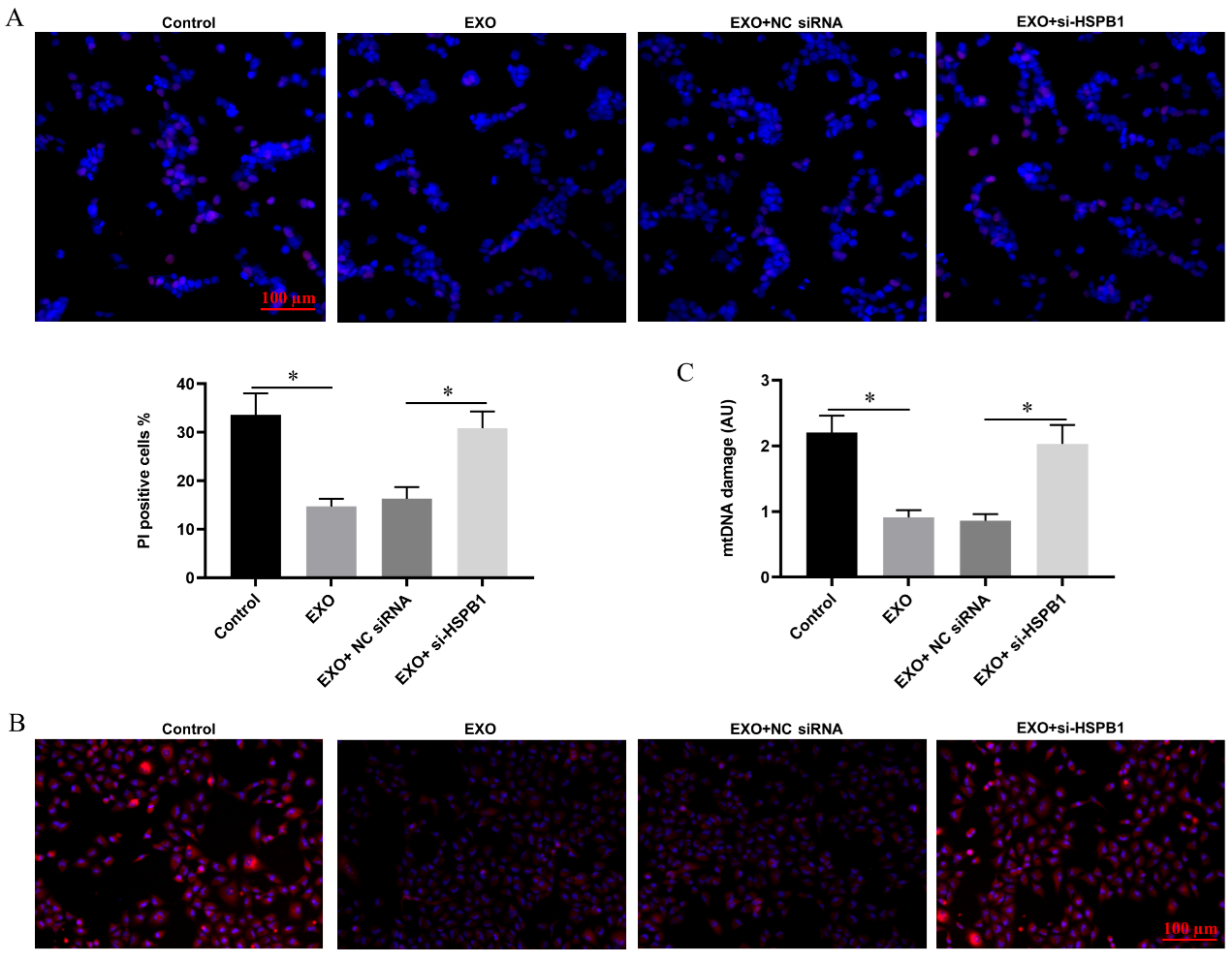


**Supplementary figure 1. The** **effect of exosomal HSPB1 on SW1990 cell death,** **lipid peroxidation and mtDNA damage.** 10 μg of exosomes was used to treat SW1990 cells alone or together with 60 nM HSPB1 siRNA (si-HSPB1), and the cells were cultured under a mildly hypoxic condition (5% O_2_-5% CO_2_). After 48 h, the cells were harvested. A. PI staining was used to evaluate cell death. B. FerroOrange was used to evaluate the level of lipid peroxide. C. mtDNA damage was assayed with a Mitochondrial DNA Damage Detection Kit. 200×. *P<0.01.


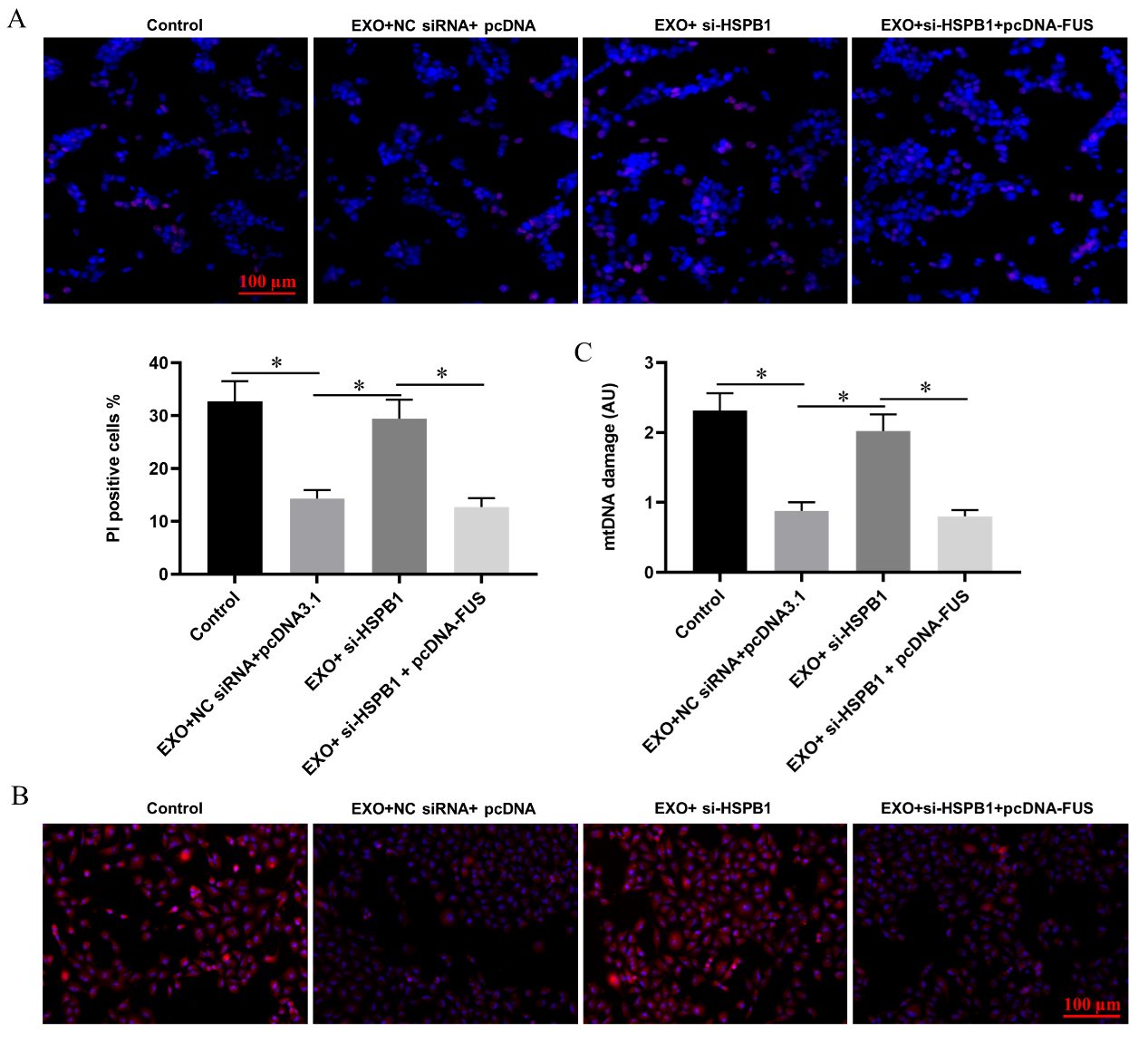


**Supplementary figure 2. The involvement of FUS in exosomal mediated suppression cell death,** **lipid peroxidation and mtDNA damage.** Exosome-incubated mildly hypoxic SW1990 cells were transfected with si-HSPB1 or together with pcDNA-FUS. A. PI staining was used to evaluate cell death. B. FerroOrange was used to evaluate the level of lipid peroxide. C. mtDNA damage was assayed with a Mitochondrial DNA Damage Detection Kit. 200×. *P<0.01.


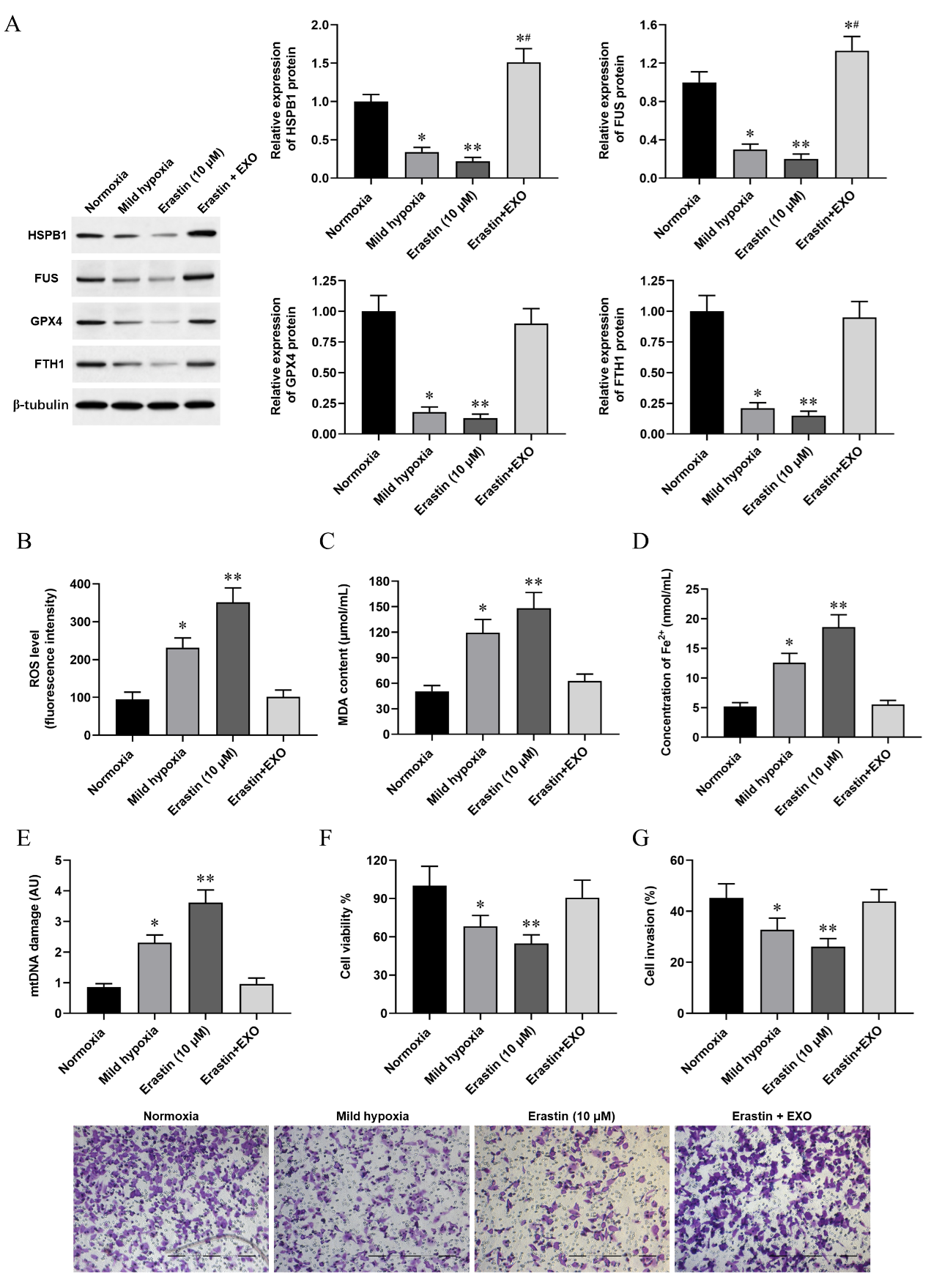


**Supplementary figure 3. Exosome treatment reversed erastin-induced ferroptosis and suppression of proliferation and invasion.** SW1990 cells were respectively treated with mild hypoxia, 10 μM erastin, and 10 μM erastin together with 10 μg of exosomes. After 48 h, the cells were harvested. A. The expression of HSPB1, FUS, GPX4 and FTH1 proteins was detected by Western blotting. B. Flow cytometry was used to detect the content of ROS. C and D. The contents of MDA and Fe2+ were detected by using corresponding kits. E. mtDNA damage was assayed with a Mitochondrial DNA Damage Detection Kit. F. Cell proliferation was analyzed by CCK-8 assay. G. The invasion ability of cells was detected by Transwell invasion assay (200×). *P<0.01, **P<0.001, compared with normoxia; ^#^P<0.01 compared with erastin.
